# A Novel Signaling Network Essential for Regulating *Pseudomonas aeruginosa* Biofilm Development

**DOI:** 10.1371/journal.ppat.1000668

**Published:** 2009-11-20

**Authors:** Olga E. Petrova, Karin Sauer

**Affiliations:** Department of Biological Sciences, Binghamton University, Binghamton, New York, United States of America; University of California San Francisco, United States of America

## Abstract

The important human pathogen *Pseudomonas aeruginosa* has been linked to numerous biofilm-related chronic infections. Here, we demonstrate that biofilm formation following the transition to the surface attached lifestyle is regulated by three previously undescribed two-component systems: BfiSR (PA4196-4197) harboring an RpoD-like domain, an OmpR-like BfmSR (PA4101-4102), and MifSR (PA5511-5512) belonging to the family of NtrC-like transcriptional regulators. These two-component systems become sequentially phosphorylated during biofilm formation. Inactivation of *bfiS*, *bfmR*, and *mifR* arrested biofilm formation at the transition to the irreversible attachment, maturation-1 and -2 stages, respectively, as indicated by analyses of biofilm architecture, and protein and phosphoprotein patterns. Moreover, discontinuation of *bfiS*, *bfmR*, and *mifR* expression in established biofilms resulted in the collapse of biofilms to an earlier developmental stage, indicating a requirement for these regulatory systems for the development and maintenance of normal biofilm architecture. Interestingly, inactivation did not affect planktonic growth, motility, polysaccharide production, or initial attachment. Further, we demonstrate the interdependency of this two-component systems network with GacS (PA0928), which was found to play a dual role in biofilm formation. This work describes a novel signal transduction network regulating committed biofilm developmental steps following attachment, in which phosphorelays and two sigma factor-dependent response regulators appear to be key components of the regulatory machinery that coordinates gene expression during *P. aeruginosa* biofilm development in response to environmental cues.

## Author Summary

Biofilms are complex communities of microorganisms encased in a matrix and attached to surfaces. It is well recognized that biofilm cells differ from their free swimming counterparts with respect to gene expression, protein production, and resistance to antibiotics and the human immune system. However, little is known about the underlying regulatory events that lead to the formation of biofilms, the primary cause of many chronic and persistent human infections. By mapping the phosphoproteome over the course of *P. aeruginosa* biofilm development, we identified three novel two-component regulatory systems that were required for the development and maturation of *P. aeruginosa* biofilms. Activation (phosphorylation) of these three regulatory systems occurred in a sequential manner and inactivation arrested biofilm formation at three distinct developmental stages. Discontinuation of *bfiS*, *bfmR*, or *mifR* expression after biofilms had already matured resulted in disaggregation/collapse of biofilms. Furthermore, this regulatory cascade appears to be linked via BfiS-dependent GacS-phosphorylation to the previously identified LadS/RetS/GacAS/RsmA network that reciprocally regulates virulence and surface attachment. Our data thus indicate the existence of a previously unidentified regulatory program of biofilm development once *P. aeruginosa* cells have committed to a surface associated lifestyle, and may provide new targets for controlling the programmed differentiation process of biofilm formation.

## Introduction

Biofilms are composed of microorganisms attached to a solid surface and encased in a hydrated polymeric matrix of their own synthesis. Biofilms form when bacteria adhere to surfaces in moist environments. Biofilm-associated microorganisms have been shown to colonize a wide variety of medical devices and have been implicated in over 80% of chronic inflammatory and infectious diseases including chronic otitis media, native valve endocarditis, gastrointestinal ulcers, urinary tract and middle ear infections, and chronic lung infections in cystic fibrosis (CF) patients [1],[2]. The human pathogen *Pseudomonas aeruginosa* is considered one of the primary causes of mortality in patients with CF, the most common life-threatening hereditary disease in Caucasians [3],[4]. In addition, *P. aeruginosa* causes a variety of diseases in individuals predisposed to infections as the result of severe burns, wounds, urinary tract or corneal injury, or immunocompromised status [5]–[8].

Biofilm cells differ from their planktonic counterparts in the genes and proteins that they express, resulting in distinct phenotypes including altered resistance to antibiotics and the human immune system [2],[9],[10]. Thus, it is not surprising that biofilms are considered to be differentiated communities compared to their planktonic counterparts [9],[11]. This is supported by the finding that various microorganisms, including *P. aeruginosa* have been shown to form biofilms in a stage-specific and coordinated manner. Biofilm formation is initiated with surface attachment by planktonic bacteria, followed by formation of clusters and microcolonies and subsequent development of differentiated structures in which individual bacteria as well as the entire community are surrounded by exopolysaccharides. The biofilm developmental cycle comes full circle when biofilms disperse [12],[13].

This process has been shown to be governed by the activities of regulatory networks that coordinate the temporal expression of various motility, adhesion, and exopolysaccharide genes in response to inter- and intracellular signaling molecules and environmental cues. Vallet et al. [14] described a transcriptional regulator MvaT in *P. aeruginosa* that represses the expression of *cup* genes involved in the chaperone-usher fimbrial assembly pathway. MvaT deletion mutants exhibited enhanced attachment. In contrast, type IV pili and flagella deletion mutants exhibited reduced attachment indicating that attachment and biofilm formation are mediated by extracellular appendages [12], [15]–[17]. Furthermore, the intracellular signaling molecule bis-(3′–5′)-cyclic diguanylic guanosine monophosphate (cyclic-di-GMP), first described to control extracellular cellulose biosynthesis in *Acetobacter xylinum* [18],[19], has been demonstrated in several microorganisms to modulate biofilm formation via the production of exopolysaccharides or matrix components, control auto-aggregation of planktonic cells, and regulate swarming motility [20]–[32]. In *P. aeruginosa*, at least two pathways have been identified to modulate cyclic-di-GMP and thus, biofilm formation. These are the *wsp* chemosensory signal transduction pathway [25] and a genetic pathway composed of the phosphodiesterase BifA, the inner membrane-localized diguanylate cyclase SadC and the cytoplasmic protein SadB [20],[21],[33]. Both are involved in the reciprocal cyclic-di-GMP-dependent regulation of Pel and Psl exopolysaccharide production as *P. aeruginosa* transitions from a planktonic to a surface associated lifestyle. Both Pel and Psl exopolysaccharides contribute to the overall architecture of biofilms and are essential for surface interaction and biofilm initiation [34],[35].

Expression of the *pel* and *psl* genes is coordinated by the global regulator RetS, a hybrid sensor kinase-response regulator protein, that plays a key role in the reciprocal regulation of virulence factors and biofilm formation required for acute versus chronic infection [36]. RetS belongs to the family of two-component regulatory systems (TCS) which translate external signals into adaptive responses by a variety of mechanisms, including control of gene expression and methylation of target proteins. RetS is postulated to act in concert with two other TCS sensor kinase-response regulator hybrids, GacS and LadS, to coordinate the expression of determinants involved in biofilm formation and the production of determinants required for cytotoxicity in *P. aeruginosa* via the small regulatory RNA *rsmZ* [36],[37]. Inactivation of RetS results in reduced cytotoxicity but increased attachment and biofilm formation, while inactivation of both LadS and GacS results in increased virulence but decreased biofilm formation capacity [36],[37]. This multi-component switch thus orchestrates the transition from the planktonic to the biofilm mode of growth by *P. aeruginosa* via phosphorylation events of the two-component regulatory system GacA/GacS [36]–[38]. Overall, the findings suggest that the transition to a surface associated lifestyle proceeds via several pathways, probably in response to environmental cues or signals present during attachment, and involves the coordinated transduction of phosphorylation events via two-component regulatory systems (TCS). This raises the question of whether the transition to later stages of biofilm formation, which coincide with distinct phenotypes compared to planktonic and initial attached bacterial cells, also involves sensing of environmental signal(s) and requires the coordinated transduction of phosphorylation events (phosphorelays).

Here we demonstrate that *P. aeruginosa* exhibits distinct protein phosphorylation patterns at various stages of biofilm development. Furthermore, we report the identification of three novel two-component regulatory systems named BfiRS (PA4196-4197), BfmRS (PA4101-4102), and MifRS (PA5511-5512) that coordinate phosphorylation events required for the progression of *P. aeruginosa* biofilm development in a stage-specific manner. These systems together form a coordinated signaling network that regulates three committed steps of the *P. aeruginosa* biofilm life cycle, in particular the transition to three later biofilm developmental stages following initial attachment, namely initiation of biofilm formation (BfiRS), biofilm maturation (BfmRS), and microcolony formation (MifRS).

## Results

The formation of biofilms has been proposed to be controlled in response to environmental signals [39]. Given that protein phosphorylation is a common modification system used in signal transduction that changes the function of proteins in response to environmental stimuli [38], we chose a phosphoproteomic approach for the detection and identification of regulatory pathways active following the transition to the surface attached mode of growth.

### Detection of differentially phosphorylated proteins over the course of biofilm formation

While phosphoproteomic analyses have become widespread in studies of regulation, signaling, development, the characterization of bacterial species and host responses during pathogenesis [40]–[49], only a limited number of studies have demonstrated that bacterial phosphoproteomes are dynamic [44],[46],[48]. We therefore used a combination of 2D/PAGE and immunoblot analysis using commercially available anti-phospho Ser/Thr antibodies (see Suppl. Fig. S1A-B for an example) to probe for the presence of signal transduction events that occur over the course of biofilm formation. Immunoblots of whole cell extracts obtained from planktonic cells and biofilm cells representing five developmental stages (reversibly and irreversibly attached cells, maturation-1 and -2 and dispersion stage; following 8, 24, 72, 144, and 216 hr of growth, respectively, see [12],[50] for timing of biofilm stages) were thus analyzed for the presence of planktonic- and biofilm-specific phosphorylation events.

**Figure 1:**
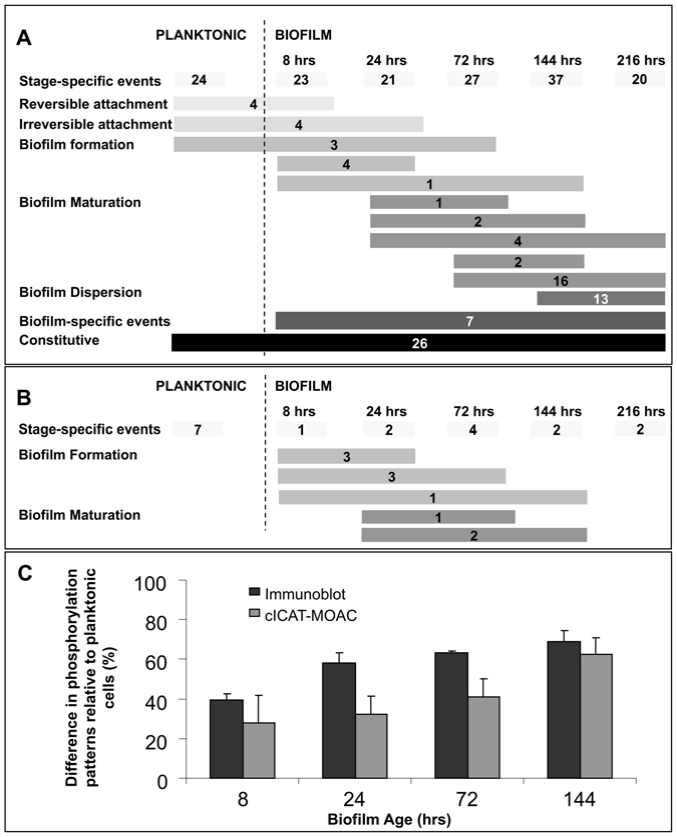
Detection of proteins phosphorylated in a stage-specific manner during biofilm development. (A) Protein phosphorylation and (B) dephosphorylation events. (A, B) Protein extracts obtained from planktonic and biofilm cells following 8, 24, 72, 144, and 216 hr of growth under flowing conditions. Proteins were first separated by 2D/PAGE and subsequently subjected to immunoblot analysis. Phosphorylation events are subcategorized according to their appearance over the course of biofilm development as being stage-specific (phosphorylation events that were only detected at one growth stage, e.g. planktonic cells or 8 h old biofilms), biofilm-specific (protein phosphorylation only detected in biofilm cells regardless of their age), and constitutive (present independent of growth condition or biofilm age). Furthermore, protein phosphorylation events that occurred at different stages over the course of biofilm formation are subcategorized as reversible and irreversible attachment, biofilm formation and maturation depending on when and for how long protein phosphorylation was detected. The values on each bar indicate the number of protein phosphorylation events detected per (sub)category. (C) Protein phosphorylation patterns over the course of biofilm development. Phosphoproteomes of *P. aeruginosa* PAO1 biofilms at different stages of development were analyzed using [i] immunoblotting of 2D gels and [ii] cleavable isotope-coded affinity tag (cICAT) mass spectrometric analysis of metaloxide affinity-enriched phosphoproteins. Biofilm protein phosphorylation patterns are shown as percent difference relative to the PAO1 planktonic phosphorylation patterns. Error bars indicate one standard deviation based on triplicate experiments.

The planktonic mode of growth coincided with 24 phosphorylated proteins that were not phosphorylated following the transition of *P. aeruginosa* to surface-associated mode of growth (Fig. 1A, stage-specific events). Additional stage-specific events were detected for biofilms differing in age. For instance, 8 hr and 24 hr old biofilms displayed 23 and 21 phosphorylation events, respectively, not detected at any other stage. Regardless of the biofilm developmental stage, 7 phosphorylation events were detected that were absent in planktonic cells (Fig. 1A, biofilm-specific events). In both modes of growth, 26 proteins were constitutively phosphorylated. In addition to biofilm stage-specific phosphorylation of proteins, protein phosphorylation events were detectable at more than one biofilm growth stage indicating that the transition to surface-associated growth coincides with distinct protein phosphorylation and dephosphorylation events. As shown in Fig. 1A, these phosphorylation events are subcategorized as occurring during the reversible and irreversible attachment, biofilm formation and maturation stage depending on when and for how long protein phosphorylation was detected. For instance, four proteins were phosphorylated both in planktonic and reversible attached cells (8 hr biofilms) but not at any other biofilm stage (Fig. 1A, reversible attachment) while 4 different proteins were phosphorylated only in planktonic cells and biofilm cells after 8 hr and 24 hr of growth under flowing conditions (Fig. 1A, irreversible attachment).

Furthermore, evidence of proteins being dephosphorylated over the course of biofilm formation was detected. Multiple proteins were found to be dephosphorylated at either a single or at multiple stages over the course of biofilm formation and maturation (Fig. 1B). Moreover, the similarity of the biofilm phosphoproteome to the planktonic phosphorylation patterns decreased from 59% in 8-hr-old biofilms to 35% in 144-hr-old, mature biofilms. The reduced similarity in phosphorylation events between biofilms and planktonic cells was mainly due to biofilm specific phosphorylation events detected at one or more stages of development. Dispersion-stage biofilms (216-hr-old) shared 43% similarity with the phosphorylation patterns of planktonically-grown *P. aeruginosa* cells (not shown). The increase in similarity between the planktonic and the 216-hr-old biofilm phosphoproteomes is consistent with previous reports indicating that cells within dispersion-stage biofilms are returning to the planktonic mode of growth [12],[50].

Protein phosphorylation in bacteria is not restricted to serine and threonine amino acid residues; however, the analysis of phosphorylation events by immunoblotting is limited to the availability of anti-phospho Ser/Thr (and tyrosine) antibodies. We therefore also purified phosphorylated proteins using metal oxide affinity chromatography (MOAC, see Fig. S1), a gel-independent approach allowing for the enrichment of phosphoproteins independent of the phosphorylation site with an up to 100% specificity [51],[52], followed by cleavable isotope coded affinity tag (cICAT) labeling and analysis by liquid chromatography tandem mass spectrometry (LC-MS/MS). This quantitative mass spectrometric approach was used to analyze protein phosphorylation patterns of biofilm cells grown to the reversible, irreversible, maturation-1 and maturation-2 biofilm stages (8–, 24–, 7–2, and 144-hr-old biofilms, respectively [12]) in comparison to those of planktonic cells.

Similarly to the results obtained via immunoblot analysis, the changes in phosphorylation events over the course of biofilm development detected using LC-MS-MS analysis appeared to be stage-specific (two examples are shown in Suppl. Fig. S2), with the similarity to the planktonic patterns decreasing from 72% in 8 hr biofilms to 38% in 144 hr biofilms (Fig. 1C). The overall stage-specific (de)phosphorylation events as well as the differences in the phosphoproteome were similar to those detected by immunoblot analysis using anti-Ser/Thr antibodies. This is the first description of the dynamic changes of the phosphoproteome occurring during biofilm development. The combination of approaches used here has not been previously used to identify phosphorylated proteins in biofilms.

### Identification of regulatory proteins involved in sensing environmental signals associated with planktonic and biofilm growth

The quantitative mass spectrometric approach by LC-MS/MS allowed for the simultaneous determination of peptide amino acid sequences by collision-induced dissociation (CID) in the MS/MS mode. Examples of two CID spectra are shown in Suppl. Fig. S2. Proteins that were confirmed to be phosphorylated by immunoblot analysis were identified using a mass spectrometric approach as well. We thus identified 48 proteins that were differentially phosphorylated at one or more biofilm developmental stage including elongation factors, ribosomal proteins, several enzymes including reductases and GMP synthase, sigma factor RpoD (Suppl. Table S1) and 11 regulatory proteins (Table 1). The majority of regulatory proteins found to be uniquely phosphorylated during planktonic growth were transcriptional regulators, while with the exception of PA2096, all regulatory proteins found to be phosphorylated during surface attached growth were identified as belonging to two-component systems (TCS) (Table 1). Of those, the sensor/response regulator hybrid GacS and PA4197 (BfiS) were found to be phosphorylated as soon as 8 hr following attachment, and PA2096 and PA4101 (BfmR) following 24 hr of surface-associated growth (Table 1). Interestingly, PA4102 (BfmS), the cognate sensor of PA4101, was found to be phosphorylated following PA4101 phosphorylation after 72 hr of biofilm growth (Table 1). The reason for the difference in the timing of phosphorylation between sensor and response regulator is unclear. It is possible that this due to the different detection methods used. The probable TCS regulatory protein PA5511 (MifR) was phosphorylated following 72 hr of surface-associated growth. The stage-specific detection and phosphorylation of PA5511 as determined by LC-MS/MS analysis in conjunction with cICAT as well as the CID spectra of a tryptic peptide of PA5511 is shown in Suppl. Fig. S2. Neither the cognate sensory protein PA5512 nor the response regulator PA4196 were identified in this study. This may be due to detection limitation (low protein concentrations, poor protein solubility, poor ionization) and/or limitation in the number of phosphorylated proteins identified (see Suppl. Tables S1 and S2 for comparison).

**Table 1:** Identification of regulatory proteins that are differentially phosphorylated over the course of *P. aeruginosa* biofilm formation.

Phosphorylation events^a^						Protein ID		Protein Description	ID Method
Plk	Biofilm (hr)								
	8	24	72	144	216				
√	-	-	-	-	-	PA0701		probable transcriptional regulator	LC-MS/MS
√	-	-	-	-	-	PA1128		probable transcriptional regulator	LC-MS/MS
√	-	-	-	-	-	PA1504		probable transcriptional regulator	LC-MS/MS
√	-	-	-	-	-	PA2824		probable sensor/response regulator hybrid	LC-MS/MS
√	√	√	√	√	√	PA2047		probable transcriptional regulator	IP/IB
-	√	√	√	√	√	PA4197	*bfiS*	probable two-component sensor	IP/IB
-	√	√	√	√	-	PA0928	*gacS*	sensor/response regulator hybrid	IP/IB; LC-MS/MS
-	-	√	√	√	√	PA2096		probable transcriptional regulator	LC-MS/MS
-	-	√	√	√	√	PA4101	*bfmR*	probable two-component response regulator	IP/IB
-	-	-	√	√	√	PA4102	*bfmS*	probable two-component sensor	LC-MS/MS
-	-	-	√	√	√	PA5511	*mifR*	probable two-component response regulator	LC-MS/MS

a √, protein phosphorylation detected by immunoblot analysis and/or LC-MS-MS analysis in conjunction with cICAT labeling.

Plk, phosphorylation events detected in cell extracts obtained from cells grown planktonically.

IP/IB, Proteins were obtained following immunoprecipitation and 2D/PAGE (IP) while protein phosphorylation was confirmed by immunoblot analysis using anti-Phospho-(Ser/Thr)Phe antibodies (IB).

As PA4197, PA4101 and GacS were all phosphorylated by 24 hr of biofilm growth, we asked whether the three proteins are modified simultaneously or in a sequential manner. We reasoned that if protein phosphorylation occurs in sequence, inactivation of one of the proteins would potentially prevent phosphorylation of the other proteins of the phosphorelay. We therefore analyzed GacS, PA4101, and PA4197 mutant biofilm phosphorylation patterns for the presence/absence of these regulators. No evidence of phosphorylation of PA4197, PA4101, or GacS was detected in *ΔPA4197* mutant biofilm phosphorylation patterns. However, phosphorylation of both GacS and PA4197 was detected in *ΔPA4101* mutant biofilms, indicating that PA4101 phosphorylation may occur downstream of GacS and PA4197. *ΔgacS* biofilm phosphorylation patterns showed an intermediate phosphorylation phenotype with PA4197 being phosphorylated but PA4101 phosphorylation lacking (not shown). PA5511 was not detected in any of the mutant biofilms analyzed (Suppl. Fig. S3). The findings suggest that phosphorylation of regulatory proteins occurs in a sequential (but probably indirect) manner over the course of biofilm formation.

To determine whether phosphorylation coincided with *de novo* gene expression or reflected biofilm-specific patterns of posttranslational modification, RT-PCR was used. *PA4101* expression was detected to be biofilm-specific, while *PA4197* and *PA5511* were constitutively expressed regardless of the mode of growth (Suppl. Fig. S4). Similarly, *retS* and *ladS* were also constitutively expressed indicating that posttranslational modifications are essential for their activity.

### Inactivation of two-component regulatory systems that are phosphorylated in a stage-specific manner leads to altered biofilm formation

As differential and sequential phosphorylation of regulatory proteins was detected over the course of *P. aeruginosa* biofilm development, we asked whether inactivation of these regulatory proteins would alter or affect the stage-specific progression of biofilm formation. We therefore focused on biofilm-specific regulatory proteins. Since the proteins PA4101, PA4197, and PA5511 were found to be phosphorylated following 8, 24, and 72 hr of biofilm growth, respectively (Table 1), corresponding to three biofilm developmental stages [9],[12], mutants in these three genes were chosen and allowed to form biofilms for 144 hr in flow cells to test for biofilm formation defects.

**Figure 2:**
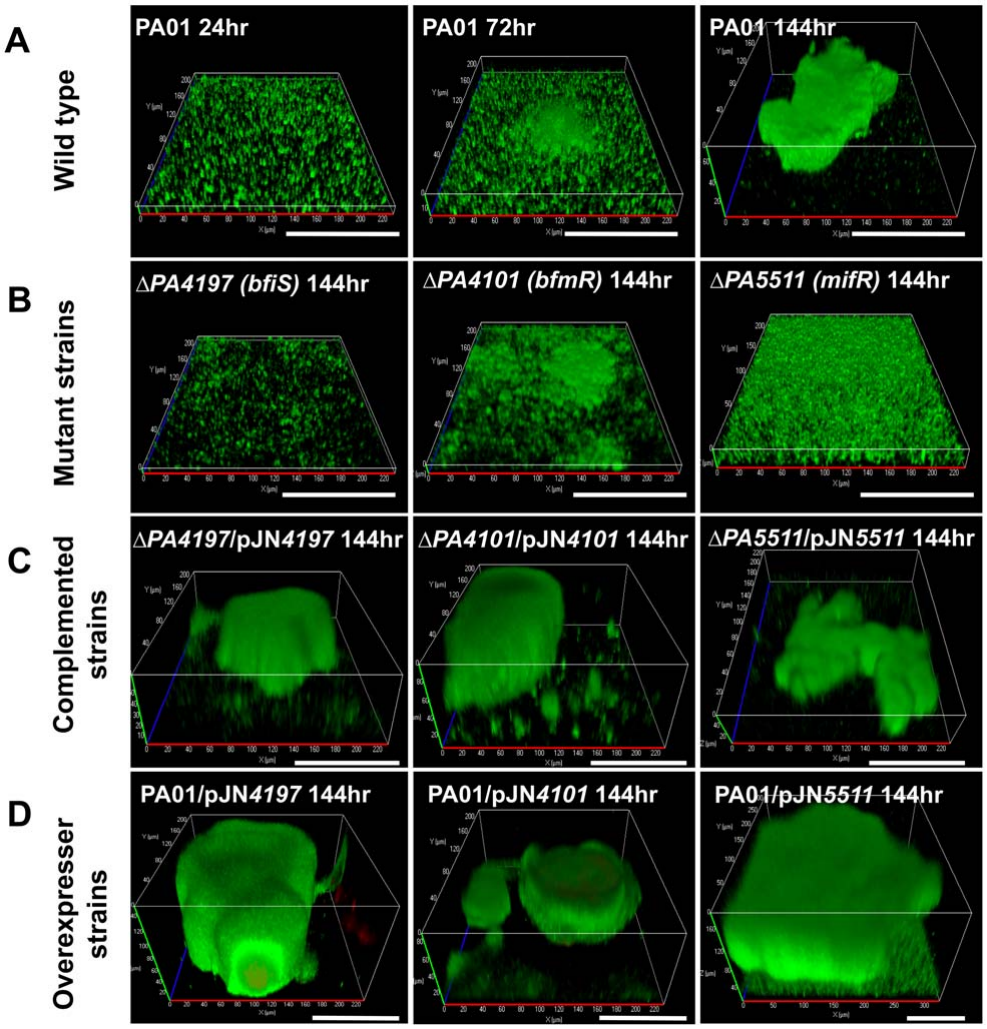
TCS mutants are arrested in biofilm development. Biofilms of strains (B) inactivated in or (C, D) overexpressing *bfiS* (PA4197), *bfmR* (PA4101), and *mifR* (PA5511), grown for 144 hours, were visualized by CSLM and compared to (A) wild type PAO1 biofilms at various stages of development. Biofilms were stained with the LIVE/DEAD *Bac*Light viability stain (Invitrogen Corp.). White bar = 100 µm.

Under the conditions tested, wild type *P. aeruginosa* biofilms reached maturity following 144 hr of growth as characterized by biofilms being composed of large microcolonies exceeding 100 µm in diameter (Fig. 2A). In contrast, PA4197 and PA4101 mutant biofilms lacked microcolonies after 144 hr of growth (Fig. 2B) and were only composed of a thin layer of cells at the substratum with an average height of 0.5 and 1.4 µm, respectively (Table 2). However, in contrast to PA4197 mutant biofilms, PA4101 mutant biofilms demonstrated the formation of some cellular aggregates which were less than 10 µm in height (Fig. 2B). Furthermore, the mutant biofilms differed significantly from wild type biofilms with respect to biomass, surface coverage, and roughness coefficient. Complementation of both PA4101 and PA4197 mutants restored biofilm formation to wild type levels (Fig. 2C, Table 2). These results allowed us to firmly conclude that the mutant biofilm phenotypes are caused by a defect in the PA4197 and PA4101 ORF.

**Table 2:** COMSTAT analysis of *P. aeruginosa* wild type and mutant biofilm structure.

Strains	Total biomass (µm^3^/µm^2^)	Substratum coverage (%)	Average thickness (µm)	Maximum thickness (µm)	Roughness coefficient
***P. aeruginosa*** ** PAO1**					
PAO1 (24 hrs)	0.95 (±0.39) 1,2	12.11 (±5.08) 1,2	0.78 (±0.38) 1,2	7.8 (±1.14) 1,2	1.69 (±0.12) 1,2
PAO1 (72 hrs)	3.27 (±1.29) 2	32.43 (±8.31)	3.02 (±1.52)	18.29 (±10.86)	1.20 (±0.31)
PAO1 (144 hrs)	7.99 (±3.63) 1	44.44 (±22.58)	7.17 (±4.97)	35.63 (±26.73)	1.24 (±0.37)
***ΔgacS*** ** mutant biofilms**					
*ΔgacS* (24 hrs)	4.01 (±3.80)	9.27 (±7.46) 1,2	4.12 (±3.87)	55 (±16.90) 1,2	1.76 (±0.15) 1,2
*ΔgacS* (72 hrs)	12.33 (±6.15) 1,2	22.01 (±13.68)	15.35 (±7.25) 1,2	82.8 (±29.45) 1,2	1.22 (±0.25)
*ΔgacS* (120 hrs)	3.47 (±2.46)	12.10 (±13.07) 1,2	4.52 (±3.24)	70.91 (±22.28) 1,2	1.65 (±0.24)
*ΔgacS* (144 hrs)	1.11 (±0.79) 1,2	3.70 (±2.13) 1,2	1.17 (±0.90) 2	11.93 (±5.16) 2	1.87 (±0.096) 1,2
*ΔgacS* (192 hrs)	1.02 (±1.00) 1,2	7.25 (±7.48) 1,2	0.91 (±0.98) 1,2	38.5 (±18.06)	1.88 (±0.12) 1,2
**Mutant biofilms following 144 hours of growth**					
*ΔPA4197 (ΔbfiS)*	1.01 (±0.44) 1,2	17.19 (±6.42) 1,2	0.51 (±0.53) 1,2	6.55 (±3.94) 1,2	1.78 (±0.19) 1,2
*ΔPA4197*/pJN4197	9.48 (±2.92)	33.05 (±11.53)	8.22 (±2.85)	44.85 (±9.17)	1.44 (±0.20)
PAO1/pJN4197	30.12 (±15.92)	31.54 (±12.23)	31.03 (±17.31)	89.78 (±29.34)	1.12 (±0.42)
*ΔPA4101* (*ΔbfmR)*	1.70 (±0.79) 1,2	15.34 (±11.88) 1,2	1.41 (±0.74) 1,2	9.06 (±5.73) 1,2	1.54 (±0.23) 1,2
*ΔPA4101/*pJN4101	7.17 (±3.04) 1	30.63 (±24.49)	6.20 (±3.58) 1	45.3 (±22.08) 1	1.42 (±0.40)
PAO1/pJN4101	18.33 (±10.49) 1,2	35.19 (±25.66)	18.15 (±10.45) 1, 2	72.69 (±22.88) 1, 2	1.17 (±0.47)
*ΔPA5511 (ΔmifR)*	3.64 (±3.20) 2	12.14 (±11.35) 1,2	3.96 (±3.43) 2	40.03 (±17.61)	1.67 (±0.29)
*ΔPA5511*/pJN5511	7.39 (±4.54)	14.43 (±7.45)	6.45 (±4.32)	42.65 (±18.49)	1.68 (±0.18)
PAO1/pJN5511	38.55 (±23.04) 1,2	39.14 (±27.30)	43.30 (±28.74) 1,2	137.55 (±43.46) 1,2	0.92 (±0.36) 1,2

COMSTAT analysis was carried out from biofilms grown in triplicate using at least 6 images per replicate.

1 Significantly different from PAO1 72 hr biofilm values (*p*≤0.05) as determined by ANOVA.

2 Significantly different from PAO1 144 hr biofilm values (*p*≤0.05) as determined by ANOVA.

Based on the role of PA4197 in the initiation of biofilm formation, we named the PA4197 ORF *B*io*f*ilm *i*nitiation *S*ensor (BfiS). BfiS is an unusual sensor that harbors a His kinase A domain typically found in two-component system (TCS) sensor proteins, a Histidine kinase-like ATPase domain involved in autophosphorylation but also in protein dephosphorylation events, and a PAS signal receiver domain [53]. The cognate response regulator BfiR (PA4196) harbors a CheY-like signal receiver domain and a LuxR-like DNA binding domain, which is also present in the quorum-sensing regulatory proteins LasR, RhlR, and QscR and in response regulators with established roles in biofilm formation (GacA, RocA1/SadA) [53]. BfiR also harbors region 4 of Sigma-70 (RpoD)-like sigma factors, a domain involved in binding to −35 promoter elements [53].

Due to its role in biofilm maturation, we named the PA4101 ORF *B*io*f*ilm *m*aturation *R*egulator (BfmR). The protein harbors an OmpR-like transcriptional regulator domain encompassing the common signal receiver and DNA-binding effector domains [53]. The cognate sensor BfmS (PA4102) is unusual in that it lacks an autophosphorylation site typically found in sensor kinases [53].

As shown in Table 1, the probable TCS regulatory protein PA5511 was phosphorylated following 72 hr of surface-associated growth. PA5511 mutant biofilms grown for 144 hr lacked clusters and microcolonies typically found in wild type biofilms following 72–144 hr of growth (Fig. 2A–B). Complementation restored biofilm formation to wild type levels (Fig. 2C, Table 2). However, when placed in a PAO1 background (PAO1*/*pJN5511), overexpression of *PA5511* resulted in biofilms composed of large microcolonies exceeding 250 µm in diameter (compared to an average cluster diameter of 150 µm in *P. aeruginosa* PAO1, Fig. 2A, D). Since cluster formation correlated with PA5511 expression levels, we named PA5511 *Mi*crocolony *f*ormation *R*egulator (MifR). MifR harbors a CheY-like receiver and a sigma-54 interaction domain [53]. The protein is on average 30–50% identical to known *P. aeruginosa* NtrC-like enhancer binding proteins including PilR, FleQ, FleR, AlgB, CbrB, and NtrC [53],[54]. The cognate sensor (MifS, PA5512) is a typical sensor kinase harboring both a His kinase A and a His kinase-like ATPase domain [53].

Since individual carbon and nitrogen sources have been demonstrated to modulate *P. aeruginosa in vitro* biofilm development and architecture [16], [55]–[58], surface motility [59] and *P. aeruginosa* cell-cell signaling (quorum sensing) [60]–[63], the biofilm architecture of all four mutant biofilms was tested using three different media including LB medium and two minimal media containing glutamate [17] or citrate [64] as sole carbon source. Under the conditions tested, the biofilm architecture of all three mutants was similar to the biofilm architecture shown in Fig. 2 independent of the media used.

### TCS mutant biofilms are arrested in the transition to later biofilm developmental stages

To determine whether the altered biofilm structure was due to arrested biofilm formation or attachment defects, we first determined whether the *P. aeruginosa* mutants are defective in attachment. Inactivation of BfiS, BfmR, and MifR (PA4197, PA4101, PA5511, respectively) did not affect initial attachment to polystyrene compared to wild type biofilms as revealed by the crystal violet microtiter plate assay and confirmed by microscopy (not shown). Furthermore, no difference in growth in broth or defect with respect to twitching, swimming, and swarming or Pel and Psl polysaccharide production was detected for any of the mutant strains (not shown). In addition, no difference in transcript abundance, as determined by semi-quantitative RT-PCR, of genes involved in Pel and Psl polysaccharide biosynthesis compared to wild type was detected (not shown). However, a *ΔgacS* mutant showed 10-fold reduced initial attachment compared to the wild type (not shown), consistent with previous findings by Parkins and colleagues [65].

These findings implied that the novel regulatory proteins were involved in the regulation of biofilm formation at later stages following initial attachment. To determine the stage at which *ΔbfiS* and *ΔbfmR* mutant biofilms were arrested, the biofilm architecture of the mutant strains after 144 hr of growth was compared to the wild type *P. aeruginosa* biofilm architecture following 24, 72, and 144 hr of growth (Table 2). Based on the comparison of 5 biofilm variables, both mutant biofilms were more similar to 24-hr-old biofilms, with *ΔbfmR* forming more substantial biofilms than *ΔbfiS* or 24 hr wild type biofilms (Table 2). Arrest of biofilm formation at the 1-day time point correlated with the timing of BfiS and BfmR phosphorylation (Tables 1–2).

Comparison of the *ΔmifR* biofilm architecture following 144 hr of growth to wild type biofilms indicated that *ΔmifR* biofilms were comparable to 72-hr-old biofilms. Since MifR was detected to be phosphorylated following 72 hr of biofilm growth (Table 1), our findings indicate that phosphorylation of MifR is essential for the progression of *P. aeruginosa* biofilms from the maturation-1 stage (72 hr) to the maturation-2 stage (144 hr).

To exclude the possibility that the *ΔbfiS*, *ΔbfmR*, and *ΔmifR* mutant biofilms may have disaggregated prematurely, the formation of mutant biofilms was monitored daily by confocal microscopy over a period of 144 hr. The *ΔbfiS* and *ΔbfmR* biofilms resembled wild type biofilms with respect to biomass and overall architecture at the 24 hr time point (see Fig. 2A–B). However, while wild type biofilms continued to mature/develop upon prolonged incubation (see Fig. 2A), no additional biomass accumulation or alteration in architecture was observed for *ΔbfiS* and *ΔbfmR* biofilms post 24 hr of growth. Furthermore, for *ΔmifR* biofilms, the progression of biofilm formation was indistinguishable from wild type *P. aeruginosa* biofilm formation for the first 72 hr of growth. However, continued incubation did not result in increased *ΔmifR* biofilm growth (biomass, thickness) or microcolony formation typically seen in wild type biofilms at the maturation-2 stage (post 72 hr of growth, Table 2, Fig. 2).

The findings clearly indicate that inactivation of these novel regulatory proteins did not cause biofilm disaggregation. Instead, our findings suggested that the mutant biofilms were incapable of progressing from the initial attachment stage to more mature biofilm stages.

### GacS plays a dual role in *P. aeruginosa* biofilm development

**Figure 3:**
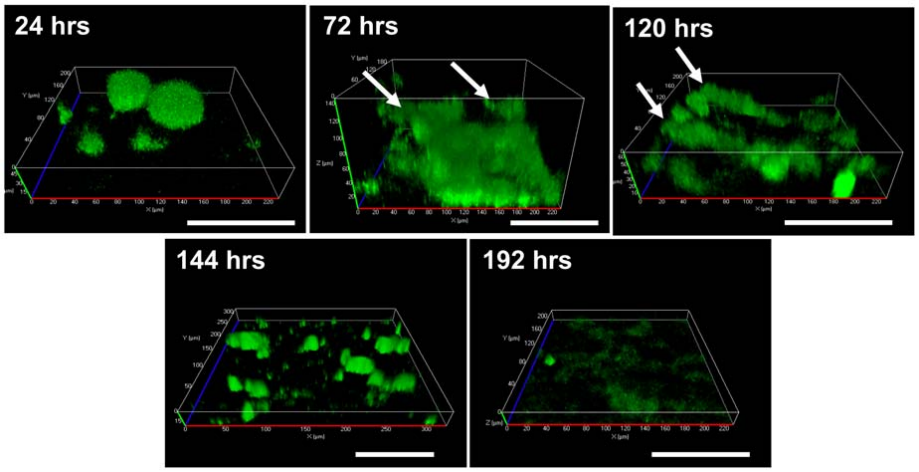
Biofilm formation by *P. aeruginosa ΔgacS*. Confocal images were acquired following 24, 72, 120, 144, and 192 hours of biofilm growth under flowing conditions. Arrows indicate non-adherent, sloughing particles. White bar = 100 µm.

Since GacS was found to be phosphorylated in a BfiS-dependent manner following 8 hrs of growth, we asked whether a *ΔgacS* mutant forms biofilms similar in architecture to *ΔbfiS* biofilms. Inactivation of *gacS* resulted in the formation of biofilms following 144 hr of growth that were similar in appearance to 24-hr-old wild type biofilms. Closer inspection of biofilm formation by *ΔgacS* over a period of 144 hr, however, indicated that the biofilm architecture (seen after 144 hr) was due to accelerated biofilm growth followed by premature disaggregation of biofilms as compared to wild type biofilms. *ΔgacS* mutant biofilms were significantly thicker than wild type biofilms following 1 and 72 hr of growth under flowing conditions, forming microcolonies and clusters exceeding 150 µm in diameter (Fig. 3). At both time points, *ΔgacS* biofilms not only exceeded the average microcolony size typically seen for wild type biofilms of the same age, but also the biomass and thickness of wild type biofilms at more mature ages (Fig. 3, Table 2). Continued growth for more than 72 hr, however, resulted in the disaggregation of *ΔgacS* mutant biofilms as indicated by the presence of large, detached clusters floating in the bulk liquid, and a significantly reduced attached biofilm biomass and biofilm thickness (Fig. 3, Table 2).

Thus, while growth of *ΔgacS* mutant biofilms following 24 hr post attachment was accelerated (Fig. 3), initial attachment was significantly reduced in this mutant (not shown). These findings suggest that GacS may play a dual role in regulating biofilm formation, which in turn may be dependent on the phosphorylation status of GacS (Table 1).

### Mutant biofilms display protein and phosphorylation patterns indicative of stage-specific arrest of biofilm development

**Figure 4:**
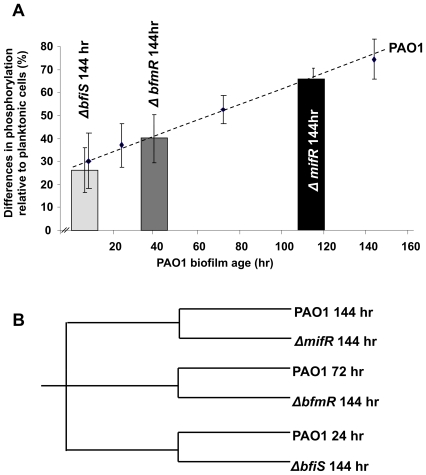
Comparison of protein phosphorylation patterns (A) and protein production patterns (B) of wild type and mutant biofilms impaired in the developmental progression. (A) The phosphoproteomes of 144-hr-old biofilms of *ΔbfiS* (light grey bar), *ΔbfmR* (dark grey bar), and *ΔmifR* (black bar) were compared to those of PAO1 biofilms following 8, 24, 72, and 144 hr of growth (black diamond, dashed line). Biofilm protein phosphorylation patterns were analyzed using a combination of immunoblotting of 2D gels and cleavable isotope-coded affinity tag (cICAT) mass spectrometric analysis of metaloxide affinity-enriched phosphoproteins and are shown as percent difference (%) relative to the planktonic phosphorylation patterns. (B) Similarity of 2D-protein production patterns as determined by Heuristic clustering. Experiments were carried out in triplicate for each strain and/or biofilm age.

Based on qualitative and quantitative analyses, BfiS (PA4197) and BfmR (PA4101) mutant biofilm architecture appeared to be the result of arrested biofilm formation following initial attachment, while inactivation of MifR (PA5511) coincided with biofilms impaired in microcolony formation at the maturation-1 stage. Since each of these biofilm developmental stages is characterized by a unique phosphorylation pattern (Figs. 1, 4, Table 1), we reasoned that if the mutant biofilms are indeed arrested in biofilm development, their phosphoproteomes will correspond to the stage at which they are arrested. We, therefore, analyzed the phosphorylation patterns of *ΔbfiS*, *ΔbfmR*, and *ΔmifR* biofilms grown for 144 hr in comparison to *P. aeruginosa* wild type biofilms grown for 8, 24, 72, and 144 hr. The phosphoproteomes were analyzed using two approaches, (i) immunoblot analysis of whole biofilm cell extracts and (ii) LC-MS/MS analysis in conjunction with cICAT labeling following MOAC purification.

The phosphoproteome of *ΔbfiS* biofilms as determined by LC-MS/MS was 74% similar (26% difference) to planktonic cells while *ΔbfmR* biofilms shared 60% of all detected phosphorylation events with planktonic cells (40% difference). This is in contrast to the phosphoproteome of 144-hr-old *P. aeruginosa* wild type biofilms, which was 62–65% different from that of planktonic cells (Fig. 4A). Furthermore, both mutant biofilms failed to exhibit phosphorylation events typically observed during normal biofilm development following 144 hr of growth (see Fig. 1, Suppl. Table S2). For instance, *ΔbfiS* and *ΔbfmR* biofilms lacked all phosphorylated proteins typically found in mature, 144-hr-old biofilms. In addition, both mutant biofilms lacked evidence for MifR phosphorylation (phosphorylated following 72 hr of wild type growth, Table 1, Suppl. Fig. S3). Instead, *ΔbfiS* biofilms exhibited stage-specific phosphorylation events typically detected in 8-hr- and 24-hr-old wild type biofilms: the Ser/Thr phosphoproteome contained 15 out of 23 phosphorylated proteins and 2 out of 21 phosphorylated proteins that are specific for 8-hr- and 24-hr-old wild type biofilms, respectively (see Fig. 1, Suppl. Table S2). Similarly, the phosphorylation patterns of *ΔbfmR* biofilms indicated the presence of 24- and 72-hr stage-specific phosphorylated proteins (not shown). The phosphorylation patterns of 144-hr-old *ΔmifR* biofilms were 62% different relative to planktonic cells, but only shared 58% similarity with mature, 144-hr-old wild type biofilms (Fig. 4A). Furthermore, *ΔmifR* biofilms exhibited 8 out of 27 maturation-1 specific protein phosphorylation events, and only 16 out of 37 maturation-2 phosphorylation events (Suppl. Table S2, see Fig. 1).

We further reasoned that if the mutant biofilms are indeed arrested in biofilm development, their whole proteomes will also correspond to the stage at which they are arrested. We therefore compared the protein production patterns of 144-hr-old *ΔbfiS*, *ΔbfmR*, and *ΔmifR* biofilms to the 2D-patterns of *P. aeruginosa* wild type biofilms grown for 24, 72 and 144 hr using 2D/PAGE, 2D ImageMaster Platinum software and heuristic clustering. As shown in Fig. 4B, cluster analysis based on protein similarity confirmed our previous findings obtained by microscopic and phosphoproteome analyses of mutant biofilms. *ΔbfiS* biofilms were more similar to 24-hr-old wild type biofilms than to wild type biofilms at more mature stages. The two protein patterns were more than 80% similar. In contrast, *ΔbfmR* biofilms were most similar to protein patterns obtained from 72-hr-old wild type biofilms (85% similarity), while those of *ΔmifR* biofilms were similar to both 72- and 144-hr-old biofilms sharing 76 and 82% similarity, respectively, to both protein patterns (Fig. 4B).

Based on analyses of biofilm architecture, as well as of protein production and phosphorylation patterns, our findings indicate that *ΔbfiS* biofilms are arrested in the transition from reversible to the irreversible attachment stage (8 hr to 24-hr-old biofilms, respectively). Inactivation of MifR appeared to result in the arrest of biofilm development in the transition between the maturation-1 and -2 stages (72 to 144 hr) while *ΔbfmR* biofilms were arrested in the transition between irreversible attachment to maturation-1 stage.

### Expression of *bfiS*, *bfmR*, and *mifR* is required for maintenance of normal biofilm architecture while loss of expression results in biofilm architecture collapse

**Figure 5:**
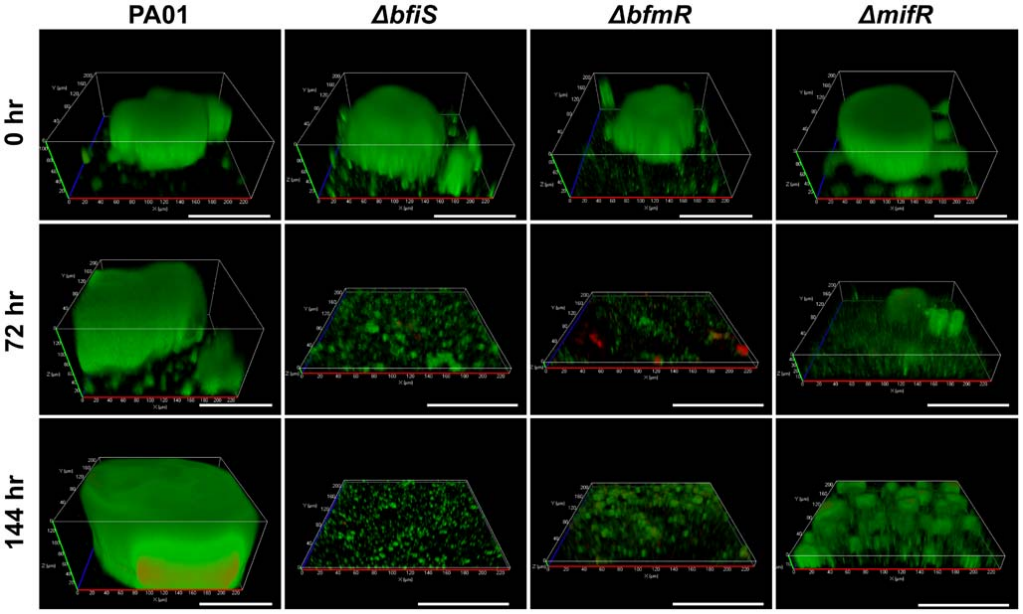
Inactivation of *bfiS* (PA4197), *bfmR* (PA4101), and *mifR* (PA5511) expression in mature biofilms results in biofilm architectural collapse and biomass loss. *P. aeruginosa* mutants complemented with plasmid-borne copies of the respective genes placed under the regulation of the arabinose-inducible P_BAD_ were grown under continuous flow conditions in glutamate minimal medium [17] in the presence of 0.1% arabinose for 144 hr after which time the biofilms were visualized by confocal microscopy (0 hr). Then, arabinose was eliminated from the growth medium and the biofilm architecture monitored post arabinose removal at the times indicated. PAO1 strain harboring the empty pJN105 vector was used as control. Biofilms were stained with the LIVE/DEAD *Bac*Light viability stain (Invitrogen Corp.). White bars = 100 µm.

Our observations indicated that BfiS (PA4197), BfmR (PA4101) and MifR (PA5511) are essential in the stage-specific development of *P. aeruginosa* biofilm formation. To determine whether these regulatory proteins are only essential during biofilm formation or are also necessary for the maintenance of established biofilms, we asked whether inactivation of these regulatory proteins in mature biofilms would affect biofilm architecture. Complemented mutant strains, harboring the respective regulator genes under the control of the arabinose-inducible P_BAD_ promoter, were allowed to grow for 144 hr in flow cells to maturity (Fig. 2C, Fig. 5–0 hr) in the presence of arabinose, after which time arabinose was removed from the growth medium to stop the transcription of the respective genes. The resulting biofilm architecture was viewed over a period of 144 hr post arabinose removal using confocal microscopy. *P. aeruginosa* wild type harboring an empty pJN105 vector was used as control.

Loss of *bfiS, bfmR*, and *mifR* expression due to arabinose removal resulted in the collapse of the mutant biofilm architecture within three days. For *ΔbfiS* and *ΔbfmR* mutant biofilms, biofilm disaggregation was noticeable as early as 24 hr post arabinose removal (not shown). The collapse was apparent by significant reduction (*P*<0.05) of biofilm variables including biofilm biomass and thickness, which further decreased upon continued incubation (Fig. 5, Table 3). Post 144 hr of arabinose removal, the biofilm architecture of the complemented mutants was similar to mutant biofilms lacking the respective regulatory gene (Figs. 2, 5). In contrast, no reduction of the wild type biofilm architecture was observed (Fig. 5, Table 3). These findings indicated that the three novel regulators are not only essential for the stage-specific progression of *P. aeruginosa* biofilms but also in the maintenance of the biofilm structure.

**Table 3:** COMSTAT analysis of *P. aeruginosa* wild type and complemented mutant biofilm structure following removal of arabinose and thus, lack of expression of PA4101, PA4197 and PA5511, respectively.

Strains	Time (hr)^a^	Total biomass (µm^3^/µm^2^)	Substratum coverage (%)	Average thickness (µm)	Maximum thickness (µm)	Roughness coefficient (dimensionless)
PAO1/pJN105	0	16.55 (±11.18)	25.42 (±12.98)	17.44 (±11.48)	75.51 (±16.60)	1.30 (±0.31)
	72	25.58 (±18.21)	32.28 (±21.77)	27.77 (±23.30)	77.22 (±32.35)	1.05 (±0.49)
	144	39.69 (±18.73)	58.99 (±19.68)	38.06 (±19.17)	76.56 (±33.66)	0.71 (±0.43)
*ΔbfiS*/pJN4197	0	18.53 (±14.12)	20.22 (±10.57)	20.45 (±16.46)	73.72 (±19.89)	1.36 (±0.39)
	72	2.94 (±2.31) *	12.63 (±7.25) *	2.81 (±2.51) *	16.61 (±7.89) *	1.56 (±0.32) *
	144	2.60 (±3.77) *	12.50 (±12.23) *	2.25 (±3.43) *	16.47 (±7.92) *	1.69 (±0.30) *
*ΔbfmR*/pJN4101	0	24.33 (±13.43)	25.03 (±13.07)	26.53 (±13.86)	79.02 (±19.30)	1.20 (±0.30)
	72	0.99 (±1.23) *	5.51 (±5.20) *	0.82 (±1.14) *	13.10 (±5.13) *	1.85 (±0.15) *
	144	4.22 (±1.88) *	24.43 (±9.14) *	3.62 (±1.80) *	13.18 (±3.17) *	1.37 (±0.25) *
*ΔmifR*/pJN5511	0	21.76 (±14.11)	35.02 (±27.26)	21.14 (±13.35)	70.57 (±20.72)	1.18 (±0.43)
	72	7.98 (±1.09) *	18.30 (±10.35) *	7.69 (±11.75) *	37.89 (±26.69) *	1.51 (±0.30) *
	144	8.70 (±3.06) *	39.63 (±8.16) *	7.98 (±2.94) *	16.99 (±8.22) *	0.93 (±0.22)

COMSTAT analysis was carried out from biofilms grown in triplicate using at least 6 images per replicate.

a Time 0 = 144-hr-old biofilms.

*: Value significantly different (*p*≤0.05) from PAO1 biofilm on corresponding day as determined by ANOVA.

## Discussion

**Figure 6:**
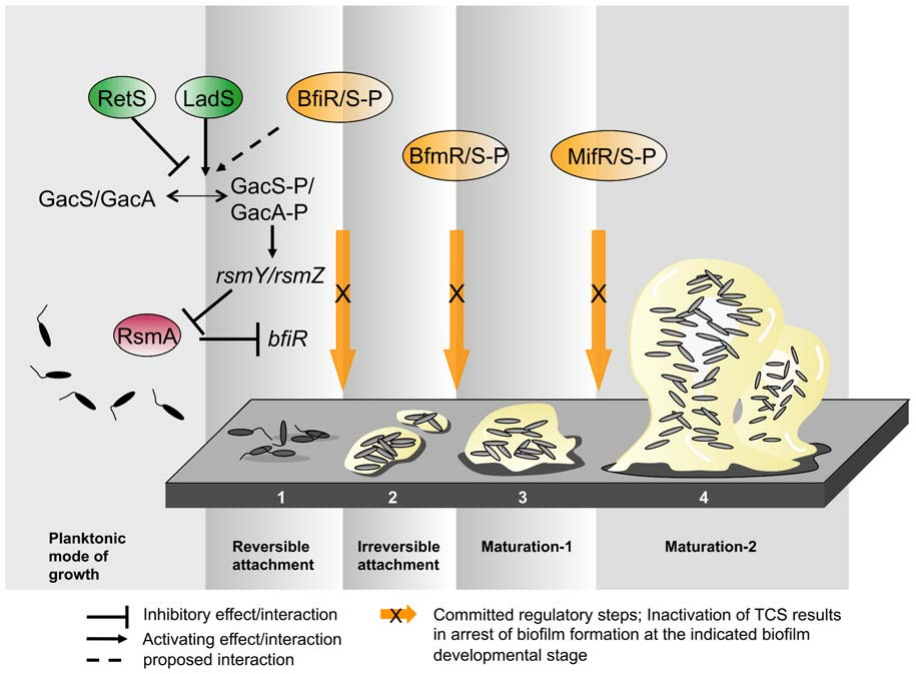
Model for the Role of novel two-component systems BfiRS, BfmRS, and MifRS in biofilm development and potential link to the multi-component signaling network LadS/RetS/GacAS/RsmA. The three novel *P. aeruginosa* two-component systems (TCS) are essential in regulating the transition to irreversible attachment (BfiRS, stage 1–2), maturation-1 (BfmRS, stage 2–3), and maturation-2 (MifRS, stage 3–4) during biofilm development in response to as of yet unknown intra- and/or extracellular signals. Phosphorylation and thus, activation occurs in a sequential manner (BfiS<GacS<BfmR/BfmS<MifR), suggesting the presence of a TCS signal transduction network during the progression of biofilm development. Furthermore, this regulatory cascade involved in stage-specific biofilm development appears to be linked, via the BfiRS system, to the LadS/RetS/GacAS/RsmA network that reciprocally regulates virulence and surface attachment. Similarly to LadS, BfiS plays a role in GacS phosphorylation. Here, GacS has been shown to play a dual role in regulating biofilm developmental steps depending on the phosphorylation status. Moreover, RsmA represses the expression of the BfiS cognate response regulator, BfiR. Adapted from [9],[77].

Evidence showing that biofilm development is a coordinated series of events coinciding with distinct phenotypes has led to the assumption that the formation of biofilms is a regulated progression [11],[12],[66]. However, biofilm development has been considered to be distinct from other developmental processes including the programmed differentiation seen in spore formation in *Bacillus subtilis* or fruiting body formation in *Myxococcus xanthus* [11], mainly because no regulatory pathways have yet been identified that are responsible for regulating committed steps in the formation of biofilms with the exception of attachment. In this study we describe the identification and initial characterization of three novel two-component systems (TCS) essential in regulating three committed steps in biofilm development. Mutation in these regulatory pathways did not affect initial attachment, motility, or Pel and Psl polysaccharide production, but instead arrested biofilm development in the transition from reversible to irreversible attachment [8 hr to 24 hr, BfiRS (PA4196-4197)], from initial attachment to the maturation-1 stage [(24 hr to 72 hr, BfmRS (PA4101-4102)], and following the maturation-1 stage [72 hr to 144 hr, MifRS (PA5511-5512)] (Fig. 6). To our knowledge, this is the first description of a regulatory program for stage-specific biofilm development.

The stage-specific arrest in biofilm formation of the mutant strains coincided with the timing of phosphorylation of the respective regulatory or sensory proteins indicating that the phosphorylation status of the three novel two-component systems is essential for their function in regulating biofilm development by *P. aeruginosa*. Furthermore, the phosphorylation of these two-component systems occurred in sequence with BfiS being phosphorylated first, followed by GacS, and lastly, MifR (Table 1, Fig. 6). The sequential phosphorylation of sensors/regulatory proteins is reminiscent of a regulatory cascade in which each phosphorylation event acts as a trigger for bacterial biofilm cells to transition to the next developmental stage (Fig. 6). Furthermore, the novel TCS systems described here appear to be linked via GacS to the multicomponent system RetS/LadS/GacAS/RsmA essential for regulating the switch between the planktonic and the sessile mode of growth. While it is not clear how the three two-component systems interact to form the observed sequential phosphorylation cascade, it is apparent from our observations that phosphorylation of each of the three novel TCS has to occur for *P. aeruginosa* biofilms to mature (Fig. 2). Possible scenarios for the sequential phosphorylation events to occur are by direct interaction or activation of a TCS system by one that is upstream in the cascade (Fig. 6), or indirectly. Since inactivation of each TCS system resulted in altered or arrested biofilms which failed to exhibit stage-specific protein production and phosphorylation events (Figs. 1, 4, Suppl. Table S2), it is likely that the mutant biofilms in turn do not produce the necessary signal(s) to activate or phosphorylate TCS system(s) that are further downstream. Thus, it is likely that inactivation of one TCS system (in)directly results in altered or arrested phosphorylation patterns and thus, lack of phosphorylation of downstream TCS systems (as observed here). Independent of the mechanism, it is evident that inactivation not only disrupts the sequence of phosphorylation events but also leads to the collapse of mature biofilms to an earlier biofilm developmental stage at which the respective regulatory proteins play a role (Fig. 5, Table 3). This is even more important as this biofilm collapse was observed under two different nutritional conditions, when grown on minimal medium using either glutamate or citrate as a sole carbon source (see also Figs. 2 and 5 for comparison of LB and glutamate minimal medium). The finding suggests that while biofilm formation, architecture and cell-cell signaling is modulated by environmental and nutritional conditions resulting in biofilm development proceeding via distinctly different pathways [16], [55]–[63], it is possible that the novel regulatory proteins identified here play a role under more than one discrete culturing condition or pathway.

The novelty of these TCS is further supported by the finding that a search for BfiS (PA4197) and BfmR (PA4101) homologues using BLAST (http://blast.ncbi.nlm.nih.gov/Blast.cgi) and BLINK (precomputed BLAST, [53]), did not reveal any proteins that have been previously characterized in the literature. However, BfiS-like sensory proteins with identities ranging between 28–68% were detected in a variety of Gram-negative bacteria, in particular in α-, β-, and γ-proteobacteria (Suppl. Table S3). No homologues, however, were detected in λ-proteobacteria or *E. coli, Klebsiella pneumoniae*, and *Enterobacter* sp. Similarly, BfmR homologues were detected among proteobacteria including *Yersinia* sp., *Burkholderia sp., Rhizobium sp.*, *Vibrio* sp, *Geobacter* sp., and *M. xanthus* with identities ranging between 50–92% (Suppl. Table S3). MifR homologues harboring a sigma-54 binding domain are present in both Gram-positive and Gram-negative bacteria including *M. xanthus* (Suppl. Table S3). The closest MifR homologue in *M. xanthus* was identified as the NtrC-like chemosensory regulator of development CrdA (48% identity). Inactivation of *crdA* has been shown to result in delayed *M. xanthus* multicellular development [67].

NtrC-like regulators belong to a family of transcriptional activators which control a variety of physiological processes in response to environmental signals [68]. This family of regulators control transcription from −12, −24 promoters recognized by RNA polymerase that utilizes the alternative sigma 54 factor encoded by *rpoN* and its analogs. At least 8 NtrC-like transcriptional regulators are involved in coordinating *M. xanthus* fruiting body formation at distinct stages of the developmental process [69]–[71]. The preponderance of developmental promoters with sigma 54 hallmarks led to the suggestion that NtrC-like activators are key components of the transcriptional machinery that coordinates gene expression during *M. xanthus* development [72]. While fruiting body formation is governed by a cascade of RpoN-dependent transcription factors in starving cells, endospore formation in *B. subtilis* requires the consecutive activity of multiple sigma factors including Sigma E, F, G, and K. Their activity is regulated by posttranslational processes, either by cleaving the precursor molecules or by sequestration of sigma factors by “anti-sigma factor” proteins in response to intercellular cues, and compartmentalization [68],[73]. Similarly, biofilm developmental processes appear to be controlled by sigma factors. Based on domain structure, two TCS regulatory proteins identified here regulate genes controlled by the sigma factors RpoD and RpoN [53],[74],[75]. BfiR harbors region 4 of Sigma-70 (RpoD)-like sigma factors, a domain involved in binding to −35 promoter elements. Activation of BfiR coincides with BfiS phosphorylation following 8 hours of surface attached growth and dephosphorylation of RpoD (Table 1). MifR harbors a sigma-54 binding (RpoN) binding domain and is dependent on the consecutive phosphorylation of BfiRS and BfmRS (see Suppl. Fig. S3). These results are consistent with the idea that biofilm development by *P. aeruginosa* is orchestrated by a regulatory cascade (Fig. 6) that is analogous to other developmental systems including spore formation in *B. subtilis* or fruiting body formation in *M. xanthus*, requiring the consecutive action of at least two sigma factors and three two-component regulatory systems in response to environmental signals.

In summary, we have evidence of three novel regulatory systems playing a role in the progression of *P. aeruginosa* biofilm development in a stage-specific manner. The only other regulatory system having been identified to play a role at later stages of biofilm formation, in particular the formation of large microcolonies and fluid-filled channels, is the three-component system SadARS (RocS1RA1), probably by controlling the expression of fimbrial *cup* genes [66],[76]. In addition, coordinated transduction of phosphorylation events via two-component systems has also been shown to play a role in attachment. A multi-component switch composed of three unusual hybrid sensor kinases, RetS, LadS, and GacS, has recently been demonstrated to reciprocally orchestrate the transition from acute to chronic infection in *P. aeruginosa*, as well as to reciprocally regulate the transition between the planktonic and biofilm modes of growth by inversely coordinating repression of genes required for initial colonization, mainly genes responsible for exopolysaccharide components of the *P. aeruginosa* biofilm matrix [36],[37]. While our study did not result in the identification of RetS or LadS, we identified GacS by two different approaches and confirmed GacS phosphorylation by immunoblot analysis (Table 1). GacS acts as a suppressor of RetS (and vice versa) with RetS regulating the suppressor activity of the membrane-bound sensor GacS by directly modulating its phosphorylation state [38]. The finding is consistent with our observation of GacS playing a dual role in biofilm formation, with phosphorylation acting as a switch in the function of GacS (Fig. 3, Table 2): GacS participates in the planktonic/biofilm switch in its non-phosphorylated state, but limits/regulates the rate of biomass accumulation and biofilm development when phosphorylated. Since phosphorylation of GacS occurred following 8 hr of surface attached growth (Table 1) and since RetS directly modulates the phosphorylation state of GacS [38], the findings may suggest that RetS only remains functional for a period of 8 hours during initial attachment after which RetS is rendered non-functional. Here, GacS was found to be phosphorylated in a BfiS dependent manner. In turn, expression of the BfiS cognate response regulator, BfiR, was found to be RsmA dependent [77] (see Fig. 6). Taken together, our observations suggest a link between the multi-component switch RetS/LadS/GacAS/RsmA which reciprocally regulates virulence and the transition between the planktonic and the surface attached mode of growth and the previously undescribed signaling network which regulates developmental steps once *P. aeruginosa* has committed to the surface associated lifestyle (Fig. 6).

Taken together, this work identifies a previously undescribed signal transduction network composed of BfiSR (PA4196-4197), BfmSR PA4101-4102), and MifSR (PA5511-5512) that sequentially regulates committed biofilm developmental steps following attachment by transcriptional and posttranscriptional mechanisms, which is linked via GacS and RsmA to the previously described multi-component switch RetS/LadS/GacAS/RsmA. Furthermore, the finding of sequential and essential regulatory steps in biofilm formation and the involvement of at least two sigma factors suggests that biofilm development is analogous to other programmed developmental processes. However, in contrast to known developmental processes, our findings suggest that both two-component regulatory systems and sigma factor dependent response regulators are key components of the transcriptional and regulatory machinery that coordinate gene expression during *P. aeruginosa* biofilm development.

## Materials and Methods

### Bacterial strains, plasmids, media, and culture conditions

All bacterial strains and plasmids used in this study are listed in Table 4. The parental strain for all studies was *P. aeruginosa* PAO1. All planktonic strains were grown in minimal medium containing glutamate as sole carbon source [17] at 22°C in shake flasks at 220 rpm. Biofilms were grown as described below at 22°C in minimal medium. In addition, biofilms were grown in VBMM medium containing citrate as sole carbon source [64] and 1/20 diluted Lennox Broth (LB). Complementation experiments were carried out in minimal medium [17] with or without 0.1% arabinose. Antibiotics were used at the following concentrations: 50–75 µg/ml gentamicin (Gm) and 50 µg/ml tetracycline (Tet) for *P. aeruginosa*; 20 µg/ml Gm, 50 µg/ml ampicillin (Ap), and 25 µg/ml kanamycin (Km) for *E. coli.*

**Table 4:** Bacterial strains and plasmids.

Strains/Plasmids		Relevant genotype or description	Source
**Strains**			
*E. coli*			
DH5α		*F^−^ φ80lacZΔM15 Δ (lacZYA-argF)U169 recA1 endA1 hsdR17(rk^−^, mk^+^)*	Invitrogen
		*phoA supE44 thi-1 gyrA96 relA1 tonA*	
*P. aeruginosa*			
PAO1		Wild type	B.H. Holloway
ΔbfiS	**	PAO1; PA4197::IS*lacZ*; Tet^R^	[84]
* Δbfm*R		PAO1; PA4101::IS*lacZ*; Tet^R^	[84]
* ΔgacS*		PAO1; *gacS*(PA0928)::IS*lacZ*; Tet^R^	[84]
* ΔmifR*		PA5511 allelic replacement in PAO1 using the vector pEX18Gm-5511	This study
PAO1-*att*Tn*7*::*gfp*		PAO1 containing pUC18T-mini-Tn7T-Gm-*gfpmut3a*; Gm^R^	This study
* ΔgacS*-*att*Tn*7*::*gfp*		*ΔgacS* containing pUC18T-mini-Tn7T-Gm-*gfpmut3a*; Gm^R^	This study
PAO1/pJN105		PAO1 bearing empty pJN105 vector; Gm^R^	This study
* Δbfm*R/pJN4101		Complementation of *Δbfm*R; Tet^R^; Gm^R^, arabinose-inducible	This study
* ΔbfiS*/pJN4197		Complementation of *ΔbfiS*; Tet^R^; Gm^R^, arabinose-inducible	This study
* ΔmifR*/pJN5511		Complementation of *ΔmifR*; Tet^R^; Gm^R^, arabinose-inducible	This study
PAO1/pJN4197		Arabinose-inducible expression of *bfiS* in PAO1; Gm^R^	This study
PAO1/pJN4101		Arabinose-inducible expression of *bfmR* in PAO1; Gm^R^	This study
PAO1/pJN5511		Arabinose-inducible expression of *mifR* in PAO1; Gm^R^	This study
**Plasmids**			
pCR2.1-TOPO		TA cloning vector; Km^R^; Ap^R^	Invitrogen
pRK2013		Helper plasmid for triparental mating; *mob; tra*; Km^R^	[85]
pUC18T-mini-Tn7T-Gm-gfpmut3a		Mobilizable mini-Tn7 vector for GFP tagging of Gm^s^ bacteria; Gm^R^	[86]
pEX18Gm		Allelic replacement suicide vector; pUC18 MCS; *oriT^+^*; *sacB^+^*; Gm^R^	[80]
pJN105		Arabinose-inducible gene expression vector; pBRR-1 MCS; *araC-*P_BAD_; Gm^R^	[81]
pET101D		Vector for directional cloning and high level V5/His fusion protein expression	Invitrogen
pEX18Gm-5511		pEX18Gm containing an *Eco*RI-*Hind*III *PA5511* in-frame gene replacement construct	This study
pET4101		*bfmR* subcloned into pET101D using the primer pair PA4101pETf/r	This study
pJN4101		*bfmR*-V5/His from PET4101 amplified with PA4101HisF/R primer pair and cloned into pJN105 at EcoRI/SpeI	This study
pJN4197		*bfiS* orf amplified using PA4197araF/R primer pair, cloned into pJN105 at *Eco*RI/*Xba*I	This study
pJN5511		*mifR* orf amplified using PA5511araF/R primer pair, cloned into pJN105 at *Eco*RI/*Sac*I	This study

### Biofilm formation

Biofilms were grown using a once-through continuous flow tube reactor system to obtain proteins and in flow cells to view the biofilm architecture as described previously [12],[13]. Quantitative analysis of epifluorescence microscopic images obtained from flow cell-grown biofilms was performed with COMSTAT image analysis software [78]. Initial biofilm formation was measured by using the microtiter dish assay system, as previously described [16].

### Phosphoprotein enrichment, detection by immunoblot analysis and 2D/PAGE-analysis

Preparation of crude protein extract and protein determination was carried out as previously described [50]. Phosphoproteins were enriched by metal oxide affinity chromatography (MOAC) essentially as described by Wolschin and colleagues [51]. MOAC has been demonstrated by Krüger et al. to result in up to 20-fold enrichment of phosphoproteins and to approach 100% specificity [52]. Briefly, 750 µg of cell extract were diluted with MOAC incubation buffer (30 mM MES, 0.2 M potassium glutamate, 0.2 M sodium aspartate, 0.25% Chaps, and 8 M urea) to a final volume of 1.5 ml, and subsequently incubated for 30 min at 4°C in the presence of 80 mg of aluminum hydroxide. Unbound phosphoproteins were removed by washing the aluminum hydroxide slurry with incubation buffer. Then, phosphoproteins were eluted from the slurry using 100 mM potassium pyrophosphate and 8 M urea, desalted by methanol-chloroform precipitation, and subsequently vacuum-dried. The resulting phosphoproteins were then used for 2D/PAGE [12],[17] or LC-MS-MS analysis in conjunction with cICAT labeling as described below. To probe for the presence of Ser/Thr-phosphorylated proteins, 2D-gels were blotted onto PVDF membranes (Biorad), and probed using anti-Phospho-(Ser/Thr)Phe antibodies as previously described [13]. In addition, pull-down assays were used to enrich for Ser/Thr-phosphorylated proteins as previously described [13] using anti-Phospho-(Ser/Thr)Phe antibodies (Cell Signaling Technologies, Danvers, MA).

Both protein and phosphoproteins patterns were analyzed using the 2D ImageMaster software (GE Healthcare, Piscataway, NJ). In addition, the heuristic clustering function provided by the 2D software was used for wild type and mutant biofilm protein patterns comparisons.

### cICAT labeling and protein identification by mass spectrometry

The cICAT reagent kits were obtained from Applied Biosystems (Framingham, MA) and the cICAT sample preparation procedure was performed according to the manufacturer's protocols. Phosphoproteins isolated from planktonic PAO1 cells were used as a reference and were labeled with the cICAT light reagent, while all biofilm-derived proteins were labeled with the cICAT heavy reagent. The combined samples containing the light- and heavy-tagged proteins were purified by cationic exchange, subsequently subjected to avidin affinity chromatography, and the purified cICAT-tagged peptides subjected to partial tag cleavage. Peptide analysis was performed using a QStarXL mass spectrometer (Applied Biosystems) coupled to an Agilent LC system. A 5micron/300 Å Magic C18 AQ reversed-phase LC column (Michrom BioResources, Inc., Auburn, CA) was utilized with a 220 minute gradient from 2–80% acetonitrile (plus 0.1% formic acid, 0.01% trifluoracetic acid). Data dependant analysis was utilized to perform MS/MS on all ions above 500 m/z. Proteins obtained from 2D-gels were identified by MALDI-TOF mass spectrometry as previously described [12],[13] and by LC-MS-MS. For the latter, tryptic digested proteins were first separated by reverse phase chromatography (2–70% acetonitrile plus 0.1% formic acid and 0.01% trifluoracetic acid, 90 min gradient) and subsequently detected and fragmented using a QStarXL mass spectrometer (Applied Biosystems).

Determination of relative peptide abundances and protein identification were accomplished as previously described [12],[13] and via analyses of TOF-MS and MS/MS data using Analyst QS 1.1 software with Bioanalyst, ProID, and ProICAT packages (Applied Biosystems). Relative percent difference between two cICAT-analyzed samples was determined using the following formula: 100 U/(U+P), where U = total number of unique, unpaired peptide TOF-MS peaks detected (i.e.: peptides present in only one of the two analyzed samples), and P = total number of cICAT peptide TOF-MS peak pairs detected (i.e.: peptides present in both samples).

### Strain construction

Isogenic mutants were constructed by allelic replacement using sucrose-counter-selection as previously described [79] using the gene replacement vector pEX18Gm [80]. Complementation was accomplished by placing the respective genes under the control of an arabinose-inducible promoter in the pJN105 vector [81]. Primers used for strain construction are listed in Suppl. Table S4.

### Motility assays

Swimming, swarming, and twitching motilities were assessed in tryptone or LB medium containing 0.3%, 0.5%, and 1.0% agar, respectively, as previously described [15],[82].

### Pel and Psl polysaccharide production

Polysaccharide production was determined using the congo red (CR) binding assays as described [34] with the following modifications: Briefly, stationary phase cultures were adjusted to OD_600_ = 0.05 in LB containing 40 mg/L CR and incubated for 8 hr at 37°C with agitation after which time the cells were removed by centrifugation and the *A*_490_ of the supernatant was determined as a measurement of CR remaining in solution.

### RT-PCR

RT-PCR was carried out to determine expression of genes encoding regulatory proteins, and proteins involved in Pel and Psl polysaccharide biosynthesis in planktonic and biofilm cells using 1 µg of total RNA [50],[83]. PCR was carried out using primers listed in Suppl. Table S4. *mreB* was used as control.

### Computational analysis

Regulator homology searches and retrieval of regulator structure and conserved domain composition were accomplished using the National Center for Biotechnology Information (http://www.ncbi.nlm.nih.gov) and the Pseudomonas Genome Database [53].

## Supporting Information

Figure S1 Comparison of phosphoprotein enrichment and detection methods. 2D/PAGE patterns of 144-hr-old, maturation-2 PAO1 biofilms were compared prior to phosphoprotein enrichment (A) and after immunoprecipitation (pull-down assay) using anti-Phospho-(Ser/Thr) antibodies (B), MOAC enrichment (C), and immunoblot detection of phosphoproteins using anti-Phospho-(Ser/Thr) antibodies (D). In average, 98 (±14.76) and 309 (±60.59) protein phosphorylation events were detected per growth stage using the immunoblotting and immunoprecipitation approaches, respectively, while 334 (±74.76) spots were detected on 2DE gels following MOAC phosphoprotein enrichment.
(0.17 MB PDF)

Figure S2 Stage-specific phosphorylation of PA3735 (A–B) and PA5511 (C–D) over the course of biofilm formation. (A, C) Stage-specific detection of cICAT-labeled peptides obtained from biofilms grown for 8, 24, 72, and 144 hours under flowing conditions (see C9-label) in comparison to peptides obtained from cells grown planktonically (see C0-label). (A) PA3735 was phosphorylated in planktonic cells and following surface attachment with the exception of 72-hour-old biofilms. Arrows indicate the cICAT peptide pair of PA3735. cICAT labeled peptides obtained from planktonic cells were used as controls (indicated by C0-label). (C) PA5511 was not phosphorylated in planktonic or early biofilm cells but only following 72 and 144 hours of biofilm growth. (B, D) MS/MS spectra showing amino acid sequence of respective peptide used to identify phosphorylated proteins.
(0.05 MB PDF)

Figure S3 Demonstration of PA5511 phosphorylation being dependent on BfiS and BfmR. Stage-specific detection of cICAT-labeled PA5511 peptides obtained from PAO1, *ΔbfiS* and *ΔbfmR* biofilms grown for 144 hours under flowing conditions (see C9-label) in comparison to peptides obtained from PAO1 grown planktonically (see C0-label).
(0.04 MB PDF)

Figure S4 Transcript abundance of genes encoding two-component regulatory systems in *P. aeruginosa* PAO1 grown planktonically and as a biofilm. Experiments were carried out in triplicate.
(0.04 MB PDF)

Table S1 Differentially phosphorylated proteins: Identification and detection of phosphorylation.
(0.12 MB DOC)

Table S2 Stage-specific posphorylation of proteins in *P. aeruginosa* planktonic and biofilm cells compared to 6-day old *ΔbfiS* and *ΔmifR* mutant biofilm phosphorylation patterns.
(0.74 MB DOC)

Table S3 BLINK search for potential BifS, BfmR, and MifR homologues.
(0.12 MB DOC)

Table S4 Oligonucleotide sequences used for RT-PCR, cloning, and targeted gene inactivation.
(0.04 MB DOC)
