# Supplementary figures and images for: A Novel Signaling Network Essential for Regulating Pseudomonas aeruginosa Biofilm Development
Source: PLoS Pathog. 2009 Nov 20;5(11):e1000668. doi: 10.1371/journal.ppat.1000668 (PMC2774163; doi:10.1371/journal.ppat.1000668)

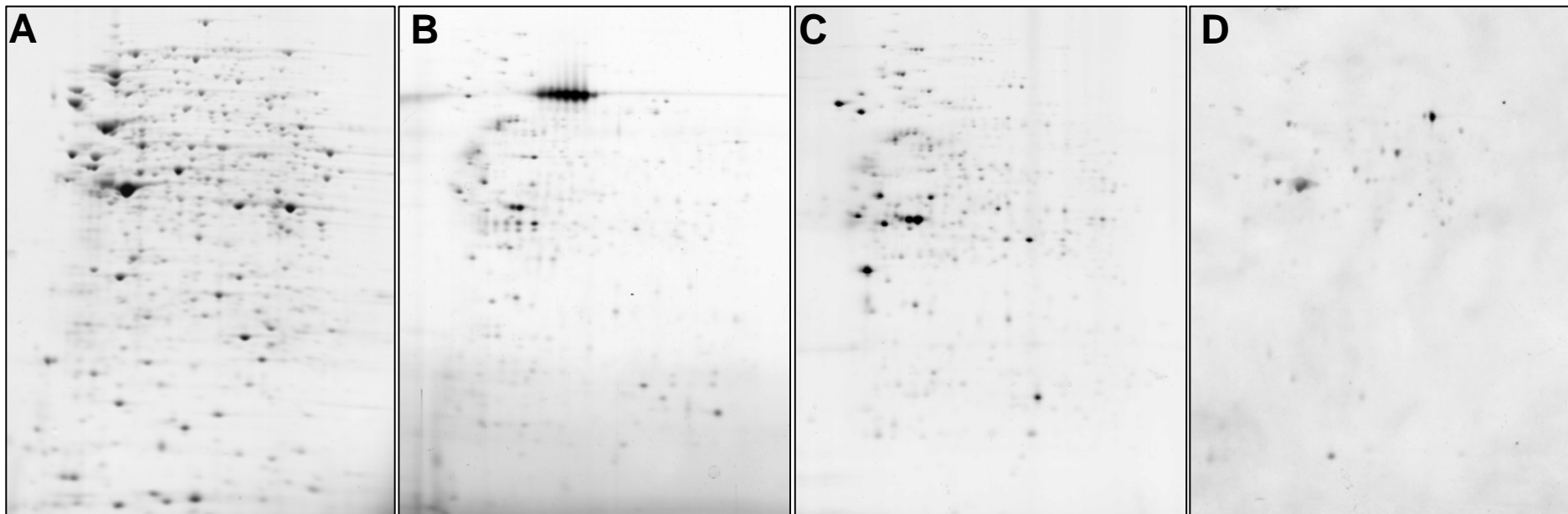

Supplement: Figure S1 — Comparison of phosphoprotein enrichment and detection methods. 2D/PAGE patterns of 144-hr-old, maturation-2 PAO1 biofilms were compared prior to phosphoprotein enrichment (A) and after immunoprecipitation (pull-down assay) using anti-Phospho-(Ser/Thr) antibodies (B), MOAC enrichment (C), and immunoblot detection of phosphoproteins using anti-Phospho-(Ser/Thr) antibodies (D). In average, 98 (±14.76) and 309 (±60.59) protein phosphorylation events were detected per growth stage using the immunoblotting and immunoprecipitation approaches, respectively, while 334 (±74.76) spots were detected on 2DE gels following MOAC phosphoprotein enrichment. (0.17 MB PDF) [file ppat.1000668.s001.pdf]

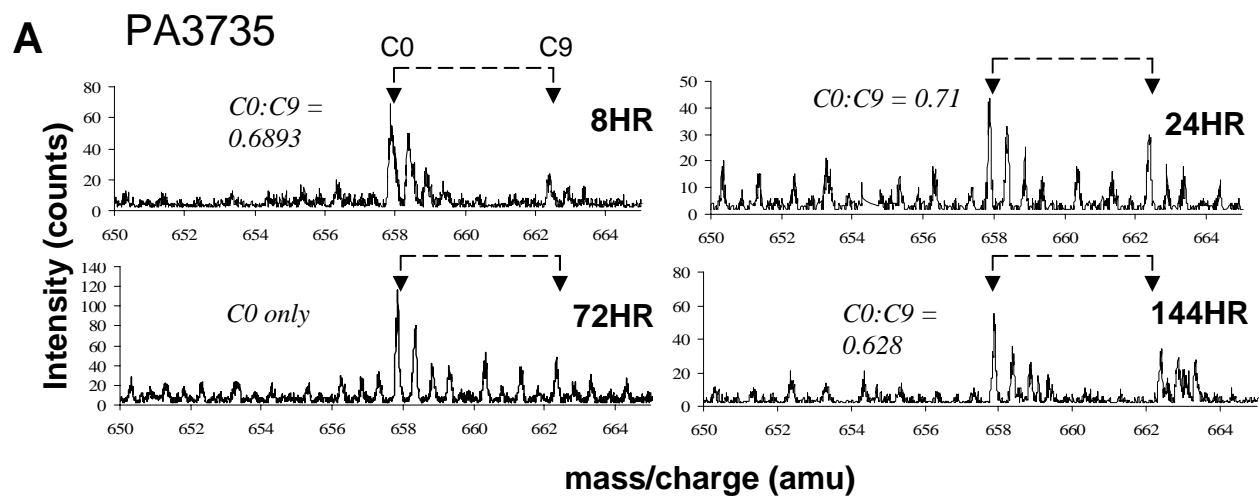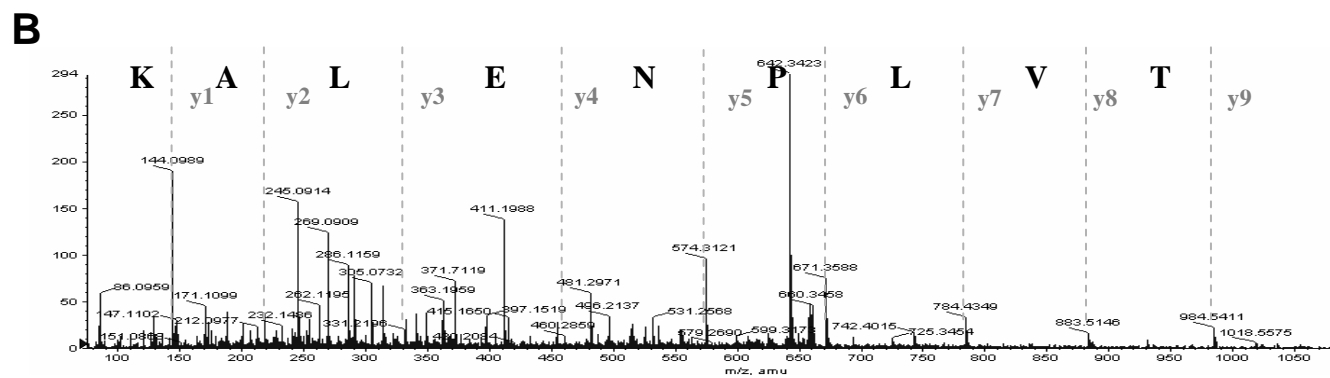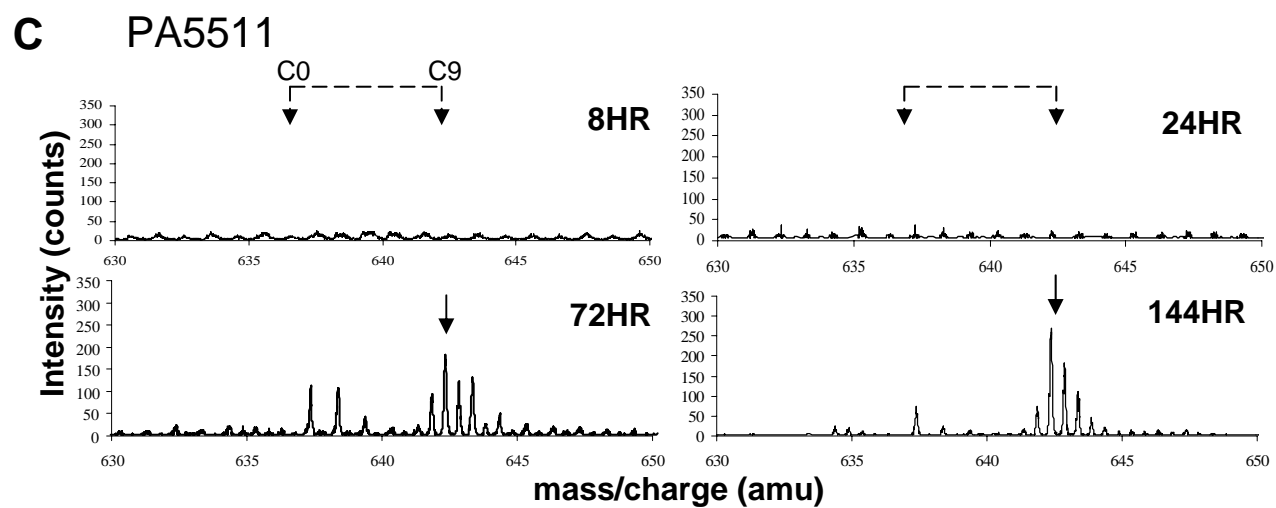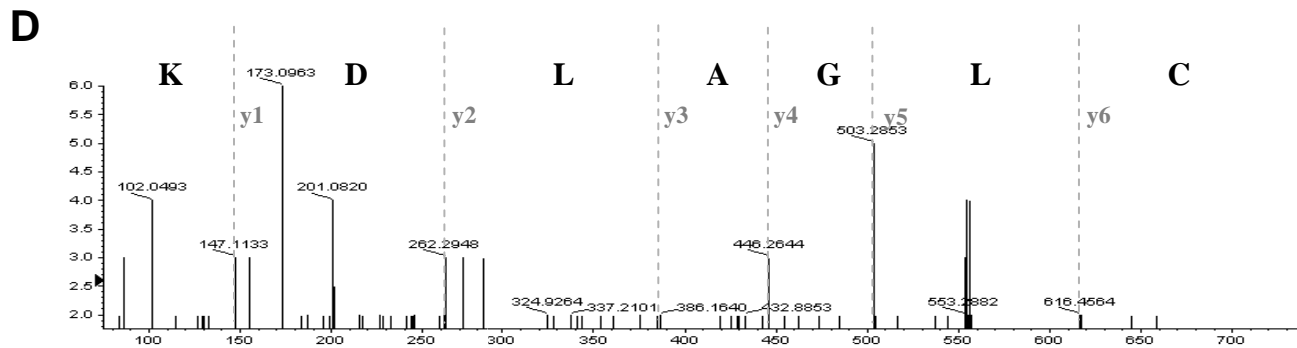

Supplement: Figure S2 — Stage-specific phosphorylation of PA3735 (A–B) and PA5511 (C–D) over the course of biofilm formation. (A, C) Stage-specific detection of cICAT-labeled peptides obtained from biofilms grown for 8, 24, 72, and 144 hours under flowing conditions (see C9-label) in comparison to peptides obtained from cells grown planktonically (see C0-label). (A) PA3735 was phosphorylated in planktonic cells and following surface attachment with the exception of 72-hour-old biofilms. Arrows indicate the cICAT peptide pair of PA3735. cICAT labeled peptides obtained from planktonic cells were used as controls (indicated by C0-label). (C) PA5511 was not phosphorylated in planktonic or early biofilm cells but only following 72 and 144 hours of biofilm growth. (B, D) MS/MS spectra showing amino acid sequence of respective peptide used to identify phosphorylated proteins. (0.05 MB PDF) [file ppat.1000668.s002.pdf]

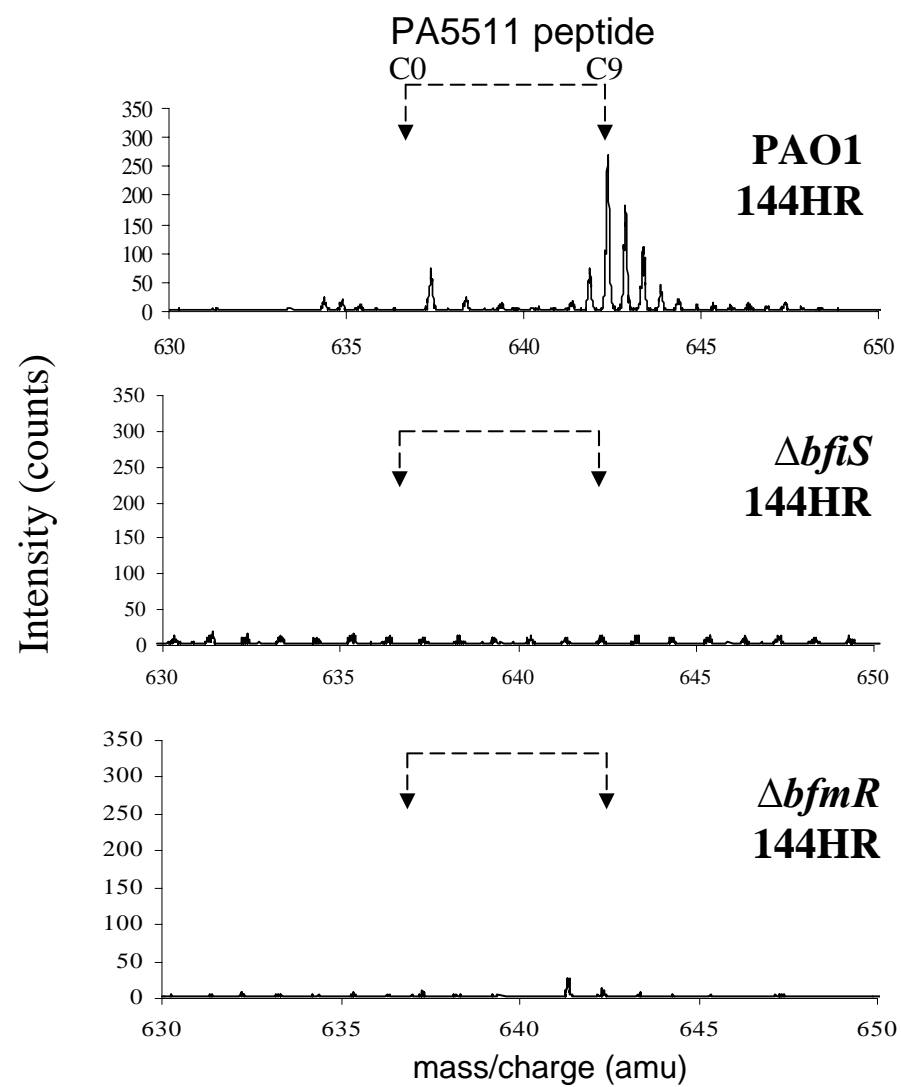

Supplement: Figure S3 — Demonstration of PA5511 phosphorylation being dependent on BfiS and BfmR. Stage-specific detection of cICAT-labeled PA5511 peptides obtained from PAO1, ΔbfiS and ΔbfmR biofilms grown for 144 hours under flowing conditions (see C9-label) in comparison to peptides obtained from PAO1 grown planktonically (see C0-label). (0.04 MB PDF) [file ppat.1000668.s003.pdf]

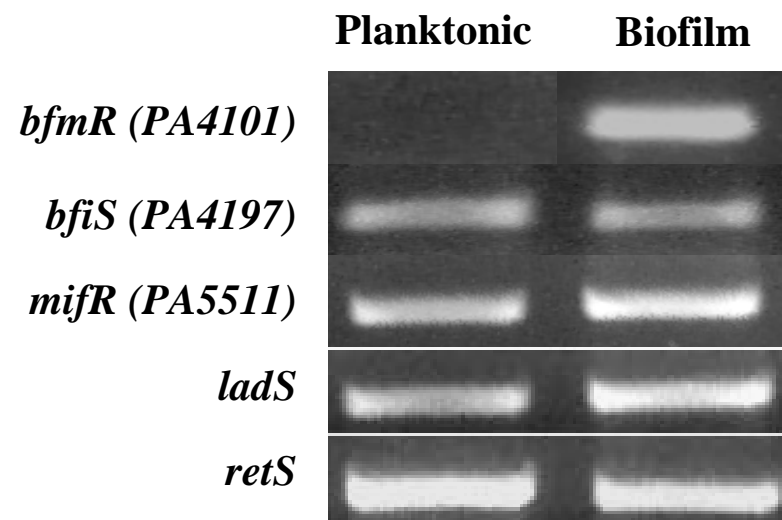

Supplement: Figure S4 — Transcript abundance of genes encoding two-component regulatory systems in P. aeruginosa PAO1 grown planktonically and as a biofilm. Experiments were carried out in triplicate. (0.04 MB PDF) [file ppat.1000668.s004.pdf]
