# Supplementary material for: A Novel Signaling Network Essential for Regulating Pseudomonas aeruginosa Biofilm Development
Source: PLoS Pathog. 2009 Nov 20;5(11):e1000668. doi: 10.1371/journal.ppat.1000668 (PMC2774163; doi:10.1371/journal.ppat.1000668)
Supplement: Table S1 — Differentially phosphorylated proteins: Identification and detection of phosphorylation. (0.12 MB DOC) [file ppat.1000668.s005.doc]

**Supplemental Table S1. Differentially phosphorylated proteins: Identification and detection of phosporylation.**

| **Phosphorylation Event^a, b^** | | | | | | **Protein ID** | |  | **ID Method^#^** |
| --- | --- | --- | --- | --- | --- | --- | --- | --- | --- |
| **PLK** | **8HR** | **24HR** | **72 HR** | **144 HR** | **216 HR** |  |  |  |  |
| √ | - | - | - | - | - | PA0139 | *ahpC* | alkyl hydroperoxide reductase subunit C | IP/IB/IB; LC-MS/MS |
| √ | - | - | - | - | - | PA0576 | *rpoD* | sigma factor RpoD | IP/IB; LC-MS/MS |
| √ | - | - | - | - | - | PA3158 | *wbpB* | probable oxidoreductase WpbB (wbpB) | LC-MS/MS |
| √ | - | - | - | - | - | PA3366 | *amiE* | aliphatic amidase | LC-MS/MS |
| √ | - | - | - | - | - | PA3533 |  | conserved hypothetical protein | LC-MS/MS |
| √ | - | - | - | - | - | PA3813 | *icsU* | probable iron-binding protein IscU (iscU) | LC-MS/MS |
| √ | - | - | - | - | - | PA5429 | *aspA* | aspartate ammonia-lyase (as) | LC-MS/MS |
| √ | √ | - | - | - | - | PA1155 | *nrdB* | tyrosyl radical-harboring component of class Ia ribonucleotide reductase | IP/IB; LC-MS/MS |
| √ | √ | - | - | - |  | PA4935 | *rpsF* | 30S ribosomal protein S6 (rpsF) | LC-MS/MS |
| √ | √ | √ | - | - | - | PA3874 | *narH* | respiratory nitrate reductase beta chain | IP/IB |
| √ | √ | √ | √ | √ | √ | PA0669 |  | probable DNA polymerase alpha chain | IP/IB |
| √ | √ | √ | √ | √ | √ | PA1464 |  | probable purine-binding chemotaxis protein | IP/IB; LC-MS/MS |
| √ | √ | √ | √ | √ | √ | PA1986 | *pqqB* | pyrroloquinoline quinone biosynthesis protein B | IP/IB |
| √ | √ | √ | √ | √ | √ | PA3513 |  | hypothetical protein | IP/IB |
| √ | √ | √ | √ | √ | √ | PA3655 | *tsf* | elongation factor Ts | IP/IB; LC-MS/MS |
| √ | √ | √ | √ | √ | √ | PA3769 | *guaA* | GMP synthase | IP/IB |
| √ | √ | √ | √ | √ | √ | PA4265PA4277 | *tufA*  *tufB* | elongation factor Tu | IP/IB; LC-MS/MS |
| √ | √ | √ | √ | √ | √ | PA4864 | *ureD* | urease accessory protein | LC-MS/MS |
| √ | √ | √ | √ | √ | √ | PA4938 | *purA* | adenylosuccinate synthetase | IP/IB |
| √ | √ | √ | √ | √ | √ | PA5322 | *algC* | phosphomannomutase AlgC | IP/IB |
| √ | √ | √ | √ | √ | √ | PA5556 | *atpA* | ATP synthase alpha chain | IP/IB |
| - | √ | √ | √ | √ | √ | PA0090 | *clpV1* | ClpV1 | IP/IB |
| - | √ | √ | √ | √ | √ | PA3049 | *rmf* | ribosome modulation factor | IP/IB |
| - | - | √ | √ | √ | - | PA5172 | *arcB* | ornithine carbamoyltransferase, catabolic | IP/IB |
|  |  |  |  |  |  |  |  |  |  |
| - | - | √ | √ | √ | √ | PA4745 | *nusA* | N utilization substance protein A | IP/IB |
| - | - | - | √ | √ | √ | PA1562 | *acnA* | aconitate hydratase 1 | IP/IB |
| - | - | - | √ | √ | √ | PA3068 | *gdhB* | NAD-dependent glutamate dehydrogenase | IP/IB |
| - | - | - | √ | √ | √ | PA5015 | *aceE* | pyruvate dehydrogenase | IP/IB |
| - | - | - | √ | √ | √ | PA5312 |  | probable aldehyde dehydrogenase | IP/IB |
| - | - | - | - | √ | √ | PA0509 | *nirN* | probable c-type cytochrome | IP/IB |
| - | - | - | - | √ | √ | PA2400 | *pvdJ* | PvdJ | IP/IB |
| - | - | - | - | √ | √ | PA2424 | *pvdL* | pvdL | IP/IB |
| - | - | - | - | √ | √ | PA4848 | *accC* | Biotin carboxylase | IP/IB |
| - | - | - | - | - | √ | PA0799 |  | probable helicase | IP/IB |
| √ | - | - | √ | √ | √ | PA4236 | *katA* | Catalase | IP/IB; LC-MS/MS |
| √ | √ | √ | - | √ | √ | PA3735 | *thrC* | threonine synthase | LC-MS/MS |
| √ | √ | √ | - | √ | √ | PA4266PA2071 | *fusA1*  *fusA2* | elongation factor G | IP/IB; LC-MS/MS |

^a^  √, protein phosphorylation detected by immunoblot analysis and/or LC-MS-MS analysis in conjunction with cICAT labeling.

^b^ PLK, planktonic cells; 8HR, 24HR, 72HR, 144HR, 216HR, biofilms grown for 8, 24, 72, 144, and 216 hours.

**^#^** IP/IB, Proteins were obtained following immunoprecipitation and 2D/PAGE (IP) while protein phosphorylation was confirmed by immunoblot analysis using anti-Phospho-(Ser/Thr)Phe antibodies (IB).
