# Supplementary material for: A Novel Signaling Network Essential for Regulating Pseudomonas aeruginosa Biofilm Development
Source: PLoS Pathog. 2009 Nov 20;5(11):e1000668. doi: 10.1371/journal.ppat.1000668 (PMC2774163; doi:10.1371/journal.ppat.1000668)
Supplement: Table S2 — Stage-specific posphorylation of proteins in P. aeruginosa planktonic and biofilm cells compared to 6-day old ΔbfiS and ΔmifR mutant biofilm phosphorylation patterns. (0.74 MB DOC) [file ppat.1000668.s006.doc]

**Supplemental Table S2. Stage-specific posphorylation of proteins in *P. aeruginosa* planktonic and biofilm cells compared to 6-day old *ΔbfiS* and *ΔmifR* mutant biofilm phosphorylation patterns.**

| **2D protein spot** | **PAO1** |  |  |  |  | | | |  | | | | | | | | ***ΔbfiS*** | | | | | | | | | | | | | | ***ΔmifR*** | | | | | | | | | | | | | **Protein Identification** | | | | | | | | | | | | | | | | | | |  |  | |
| --- | --- | --- | --- | --- | --- | --- | --- | --- | --- | --- | --- | --- | --- | --- | --- | --- | --- | --- | --- | --- | --- | --- | --- | --- | --- | --- | --- | --- | --- | --- | --- | --- | --- | --- | --- | --- | --- | --- | --- | --- | --- | --- | --- | --- | --- | --- | --- | --- | --- | --- | --- | --- | --- | --- | --- | --- | --- | --- | --- | --- | --- | --- | --- | --- | --- |
|  | **PL*** | **8HR** | **24 HR** | **72 HR** | **144 HR** | | | | **216 HR** | | | | | | | | **144HR** | | | | | | | | | | | | | | **144HR** | | | | | | | | | | | | | ***Gene ID*** | | | | | | | | **PA locus** | | | | | | **Protein ID** | | | | | |  | |
| **Differentially phosphorylated 2D-proteins** | | | | | | |  | | | | |  | | |  | | | | |  | | |  | | | |  | | | | | | | |  | | | |  | | | | |  | | | | | | | |  | | | | | |  | | | | | |  | |
| ST1 | v | v | v | v | v | | | | v | | | | | | | | v | | | | | | | | | | | | | | v | | | | | | | | | | | | |  | | | | | | | | PA2047 | | | | | | probable transcriptional regulator | | | | | |  | |
| ST10 | v | v | - | v | v | | | | v | | | | | | | | v | | | | | | | | | | | | | | v | | | | | | | | | | | | |  | | | | | | | |  | | | | | |  | | | | | |  | |
| ST11 | - | - | - | v | v | | | | v | | | | | | | | - | | | | | | | | | | | | | | v | | | | | | | | | | | | |  | | | | | | | |  | | | | | |  | | | | | |  | |
| ST12 | v | v | v | v | v | | | | v | | | | | | | | v | | | | | | | | | | | | | | v | | | | | | | | | | | | | *algC* | | | | | | | | PA5322 | | | | | | phosphomannomutase AlgC | | | | | |  | |
| ST13 | v | v | v | v | - | | | | v | | | | | | | | v | | | | | | | | | | | | | | v | | | | | | | | | | | | |  | | | | | | | |  | | | | | |  | | | | | |  | |
| ST14 | v | v | v | v | v | | | | v | | | | | | | | v | | | | | | | | | | | | | | v | | | | | | | | | | | | |  | | | | | | | | PA3513 | | | | | | hypothetical protein | | | | | |  | |
| ST15 | - | - | - | v | v | | | | v | | | | | | | | - | | | | | | | | | | | | | | v | | | | | | | | | | | | |  | | | | | | | |  | | | | | |  | | | | | |  | |
| ST16 | v | v | v | v | v | | | | v | | | | | | | | v | | | | | | | | | | | | | | v | | | | | | | | | | | | |  | | | | | | | |  | | | | | |  | | | | | |  | |
| ST17 | v | v | v | v | v | | | | v | | | | | | | | v | | | | | | | | | | | | | | v | | | | | | | | | | | | |  | | | | | | | |  | | | | | |  | | | | | |  | |
| ST18 | v | v | v | v | v | | | | v | | | | | | | | v | | | | | | | | | | | | | | - | | | | | | | | | | | | |  | | | | | | | |  | | | | | |  | | | | | |  | |
| ST19 | - | - | - | v | v | | | | v | | | | | | | | - | | | | | | | | | | | | | | - | | | | | | | | | | | | |  | | | | | | | |  | | | | | |  | | | | | |  | |
| ST2 | v | v | v | v | v | | | | v | | | | | | | | v | | | | | | | | | | | | | | v | | | | | | | | | | | | | *tufA/tufB* | | | | | | | | PA4265/PA4277 | | | | | | elongation factor Tu | | | | | |  | |
| ST20 | v | - | - | - | v | | | | v | | | | | | | | - | | | | | | | | | | | | | | v | | | | | | | | | | | | |  | | | | | | | |  | | | | | |  | | | | | |  | |
| ST21 | v | v | v | v | v | | | | v | | | | | | | | v | | | | | | | | | | | | | | v | | | | | | | | | | | | | *pqqB* | | | | | | | | PA1986 | | | | | | pyrroloquinoline quinone biosynthesis protein B | | | | | |  | |
| ST22 | v | - | - | - | - | | | | v | | | | | | | | - | | | | | | | | | | | | | | v | | | | | | | | | | | | |  | | | | | | | |  | | | | | |  | | | | | |  | |
| ST23 | - | - | - | - | v | | | | v | | | | | | | | - | | | | | | | | | | | | | | v | | | | | | | | | | | | |  | | | | | | | |  | | | | | |  | | | | | |  | |
| ST24 | - | - | - | - | v | | | | v | | | | | | | | - | | | | | | | | | | | | | | v | | | | | | | | | | | | |  | | | | | | | |  | | | | | |  | | | | | |  | |
| ST25 | - | - | - | - | v | | | | v | | | | | | | | - | | | | | | | | | | | | | | - | | | | | | | | | | | | |  | | | | | | | | PA2400 | | | | | | probable non-ribosomal peptide synthetase | | | | | |  | |
| ST26 | v | - | v | v | v | | | | v | | | | | | | | v | | | | | | | | | | | | | | v | | | | | | | | | | | | |  | | | | | | | |  | | | | | |  | | | | | |  | |
| ST27 | v | - | - | v | v | | | | v | | | | | | | | - | | | | | | | | | | | | | | v | | | | | | | | | | | | |  | | | | | | | |  | | | | | |  | | | | | |  | |
| ST28 | - | - | - | - | v | | | | v | | | | | | | | - | | | | | | | | | | | | | | v | | | | | | | | | | | | |  | | | | | | | |  | | | | | |  | | | | | |  | |
| ST29 | - | - | - | - | v | | | | v | | | | | | | | - | | | | | | | | | | | | | | - | | | | | | | | | | | | |  | | | | | | | |  | | | | | |  | | | | | |  | |
| ST3 | v | v | v | v | v | | | | v | | | | | | | | v | | | | | | | | | | | | | | v | | | | | | | | | | | | | *purA* | | | | | | | | PA4938 | | | | | | adenylosuccinate synthetase | | | | | |  | |
| ST30 | v | v | v | v | v | | | | v | | | | | | | | v | | | | | | | | | | | | | | v | | | | | | | | | | | | |  | | | | | | | |  | | | | | |  | | | | | |  | |
| ST31 | - | v | v | v | v | | | | v | | | | | | | | - | | | | | | | | | | | | | | v | | | | | | | | | | | | |  | | | | | | | | PA4197 | | | | | | probable two-component sensor | | | | | |  | |
| ST32 | v | v | v | v | v | | | | v | | | | | | | | v | | | | | | | | | | | | | | v | | | | | | | | | | | | |  | | | | | | | |  | | | | | |  | | | | | |  | |
| ST33 | v | v | v | v | - | | | | v | | | | | | | | v | | | | | | | | | | | | | | v | | | | | | | | | | | | |  | | | | | | | |  | | | | | |  | | | | | |  | |
| ST34 | - | v | v | - | - | | | | - | | | | | | | | v | | | | | | | | | | | | | | v | | | | | | | | | | | | |  | | | | | | | |  | | | | | |  | | | | | |  | |
| ST35 | v | v | v | v | v | | | | v | | | | | | | | v | | | | | | | | | | | | | | v | | | | | | | | | | | | |  | | | | | | | |  | | | | | |  | | | | | |  | |
| ST36 | - | v | v | v | v | | | | v | | | | | | | | v | | | | | | | | | | | | | | v | | | | | | | | | | | | |  | | | | | | | |  | | | | | |  | | | | | |  | |
| ST37 | v | v | - | - | - | | | | - | | | | | | | | v | | | | | | | | | | | | | | v | | | | | | | | | | | | |  | | | | | | | |  | | | | | |  | | | | | |  | |
| ST38 | v | v | - | v | v | | | | - | | | | | | | | v | | | | | | | | | | | | | | v | | | | | | | | | | | | |  | | | | | | | |  | | | | | |  | | | | | |  | |
| ST39 | v | v | - | v | v | | | | v | | | | | | | | v | | | | | | | | | | | | | | v | | | | | | | | | | | | |  | | | | | | | |  | | | | | |  | | | | | |  | |
| ST4 | v | v | v | v | - | | | | - | | | | | | | | v | | | | | | | | | | | | | | - | | | | | | | | | | | | |  | | | | | | | |  | | | | | |  | | | | | |  | |
| ST40 | - | v | v | v | v | | | | v | | | | | | | | v | | | | | | | | | | | | | | v | | | | | | | | | | | | | *clpV1* | | | | | | | | PA0090 | | | | | | ClpV1 | | | | | |  | |
| ST41 | v | v | v | v | v | | | | v | | | | | | | | v | | | | | | | | | | | | | | v | | | | | | | | | | | | |  | | | | | | | |  | | | | | |  | | | | | |  | |
| ST42 | - | v | v | v | v | | | | v | | | | | | | | v | | | | | | | | | | | | | | - | | | | | | | | | | | | |  | | | | | | | |  | | | | | |  | | | | | |  | |
| ST43 | - | - | v | v | v | | | | - | | | | | | | | - | | | | | | | | | | | | | | - | | | | | | | | | | | | | *arcB* | | | | | | | | PA5172 | | | | | | Ornithine carbamoyltransferase | | | | | |  | |
| ST44 | - | v | v | v | v | | | | - | | | | | | | | - | | | | | | | | | | | | | | v | | | | | | | | | | | | | *gacS* | | | | | | | | PA0928 | | | | | | sensor/response regulator hybrid | | | | | |  | |
| ST45 | v | - | - | v | v | | | | v | | | | | | | | v | | | | | | | | | | | | | | v | | | | | | | | | | | | | *katA* | | | | | | | | PA4236 | | | | | | catalase | | | | | |  | |
| ST46 | - | - | v | v | v | | | | v | | | | | | | | v | | | | | | | | | | | | | | - | | | | | | | | | | | | |  | | | | | | | |  | | | | | |  | | | | | |  | |
| ST47 | v | v | v | v | v | | | | v | | | | | | | | v | | | | | | | | | | | | | | v | | | | | | | | | | | | |  | | | | | | | |  | | | | | |  | | | | | |  | |
| ST48 | v | v | v | - | v | | | | v | | | | | | | | v | | | | | | | | | | | | | | v | | | | | | | | | | | | |  | | | | | | | |  | | | | | |  | | | | | |  | |
| ST49 | - | v | v | v | v | | | | v | | | | | | | | v | | | | | | | | | | | | | | v | | | | | | | | | | | | | *rmf* | | | | | | | | PA3049 | | | | | | ribosome modulation factor | | | | | |  | |
| ST5 | v | v | v | v | v | | | | v | | | | | | | | v | | | | | | | | | | | | | | v | | | | | | | | | | | | |  | | | | | | | |  | | | | | |  | | | | | |  | |
| ST50 | - | v | v | - | - | | | | - | | | | | | | | v | | | | | | | | | | | | | | v | | | | | | | | | | | | |  | | | | | | | |  | | | | | |  | | | | | |  | |
| ST51 | - | v | v | - | - | | | | - | | | | | | | | - | | | | | | | | | | | | | | v | | | | | | | | | | | | |  | | | | | | | |  | | | | | |  | | | | | |  | |
| ST52 | - | - | - | v | v | | | | v | | | | | | | | v | | | | | | | | | | | | | | - | | | | | | | | | | | | |  | | | | | | | |  | | | | | |  | | | | | |  | |
| ST53 | v | v | v | - | - | | | | - | | | | | | | | v | | | | | | | | | | | | | | - | | | | | | | | | | | | | *narH* | | | | | | | | PA3874 | | | | | | respiratory nitrate reductase beta chain | | | | | |  | |
| ST54 | v | v | v | v | v | | | | v | | | | | | | | v | | | | | | | | | | | | | | v | | | | | | | | | | | | |  | | | | | | | |  | | | | | |  | | | | | |  | |
| ST55 | - | - | - | v | v | | | | v | | | | | | | | - | | | | | | | | | | | | | | v | | | | | | | | | | | | |  | | | | | | | |  | | | | | |  | | | | | |  | |
| ST56 | v | v | v | v | v | | | | v | | | | | | | | v | | | | | | | | | | | | | | v | | | | | | | | | | | | |  | | | | | | | |  | | | | | |  | | | | | |  | |
| ST57 | - | - | - | v | v | | | | v | | | | | | | | v | | | | | | | | | | | | | | v | | | | | | | | | | | | |  | | | | | | | |  | | | | | |  | | | | | |  | |
| ST58 | - | - | - | v | v | | | | v | | | | | | | | v | | | | | | | | | | | | | | v | | | | | | | | | | | | | *acnA* | | | | | | | | PA1562 | | | | | | aconitate hydratase 1 | | | | | |  | |
| ST59 | - | - | - | v | v | | | | v | | | | | | | | v | | | | | | | | | | | | | | v | | | | | | | | | | | | | *aceE* | | | | | | | | PA5015 | | | | | | pyruvate dehydrogenase | | | | | |  | |
| ST6 | v | v | v | v | v | | | | v | | | | | | | | v | | | | | | | | | | | | | | - | | | | | | | | | | | | | *atpA* | | | | | | | | PA5556 | | | | | | ATP synthase alpha chain | | | | | |  | |
| ST60 | - | - | - | v | v | | | | v | | | | | | | | - | | | | | | | | | | | | | | v | | | | | | | | | | | | |  | | | | | | | |  | | | | | |  | | | | | |  | |
| ST61 | v | v | v | v | v | | | | v | | | | | | | | v | | | | | | | | | | | | | | v | | | | | | | | | | | | |  | | | | | | | | PA0669 | | | | | | probable DNA polymerase alpha chain | | | | | |  | |
| ST62 | - | - | - | v | v | | | | v | | | | | | | | - | | | | | | | | | | | | | | v | | | | | | | | | | | | |  | | | | | | | |  | | | | | |  | | | | | |  | |
| ST63 | - | - | v | v | v | | | | - | | | | | | | | v | | | | | | | | | | | | | | v | | | | | | | | | | | | |  | | | | | | | |  | | | | | |  | | | | | |  | |
| ST64 | v | v | v | v | v | | | | v | | | | | | | | v | | | | | | | | | | | | | | v | | | | | | | | | | | | | *guaA* | | | | | | | | PA3769 | | | | | | GMP synthase | | | | | |  | |
| ST65 | v | v | v | - | v | | | | v | | | | | | | | v | | | | | | | | | | | | | | v | | | | | | | | | | | | |  | | | | | | | |  | | | | | |  | | | | | |  | |
| ST66 | - | - | - | - | v | | | | v | | | | | | | | v | | | | | | | | | | | | | | - | | | | | | | | | | | | | *pvdL* | | | | | | | | PA2424 | | | | | | PvdL | | | | | |  | |
| ST67 | v | v | - | - | v | | | | v | | | | | | | | v | | | | | | | | | | | | | | v | | | | | | | | | | | | |  | | | | | | | |  | | | | | |  | | | | | |  | |
| ST68 | v | v | - | - | - | | | | - | | | | | | | | - | | | | | | | | | | | | | | - | | | | | | | | | | | | |  | | | | | | | |  | | | | | |  | | | | | |  | |
| ST69 | v | v | v | v | - | | | | - | | | | | | | | v | | | | | | | | | | | | | | v | | | | | | | | | | | | |  | | | | | | | |  | | | | | |  | | | | | |  | |
| ST7 | - | - | v | v | v | | | | v | | | | | | | | - | | | | | | | | | | | | | | v | | | | | | | | | | | | |  | | | | | | | |  | | | | | |  | | | | | |  | |
| ST70 | v | v | v | - | - | | | | - | | | | | | | | v | | | | | | | | | | | | | | - | | | | | | | | | | | | |  | | | | | | | |  | | | | | |  | | | | | |  | |
| ST71 | v | v | - | - | - | | | | v | | | | | | | | v | | | | | | | | | | | | | | - | | | | | | | | | | | | |  | | | | | | | |  | | | | | |  | | | | | |  | |
| ST72 | v | v | - | - | - | | | | - | | | | | | | | v | | | | | | | | | | | | | | - | | | | | | | | | | | | |  | | | | | | | |  | | | | | |  | | | | | |  | |
| ST73 | v | v | v | v | v | | | | v | | | | | | | | v | | | | | | | | | | | | | | v | | | | | | | | | | | | | *tsf* | | | | | | | | PA3655 | | | | | | elongation factor Ts | | | | | |  | |
| ST74 | v | v | v | v | v | | | | v | | | | | | | | v | | | | | | | | | | | | | | v | | | | | | | | | | | | |  | | | | | | | |  | | | | | |  | | | | | |  | |
| ST75 | v | v | - | - | - | | | | v | | | | | | | | v | | | | | | | | | | | | | | - | | | | | | | | | | | | |  | | | | | | | |  | | | | | |  | | | | | |  | |
| ST76 | v | v | v | - | - | | | | - | | | | | | | | v | | | | | | | | | | | | | | v | | | | | | | | | | | | |  | | | | | | | |  | | | | | |  | | | | | |  | |
| ST77 | v | v | v | v | v | | | | v | | | | | | | | v | | | | | | | | | | | | | | v | | | | | | | | | | | | |  | | | | | | | |  | | | | | |  | | | | | |  | |
| ST78 | v | v | v | v | v | | | | v | | | | | | | | v | | | | | | | | | | | | | | v | | | | | | | | | | | | |  | | | | | | | |  | | | | | |  | | | | | |  | |
| ST79 | - | v | v | v | v | | | | v | | | | | | | | v | | | | | | | | | | | | | | v | | | | | | | | | | | | |  | | | | | | | |  | | | | | |  | | | | | |  | |
| ST8 | v | v | v | v | v | | | | v | | | | | | | | v | | | | | | | | | | | | | | v | | | | | | | | | | | | |  | | | | | | | |  | | | | | |  | | | | | |  | |
| ST80 | v | v | v | v | v | | | | - | | | | | | | | v | | | | | | | | | | | | | | v | | | | | | | | | | | | |  | | | | | | | |  | | | | | |  | | | | | |  | |
| ST81 | v | - | - | v | v | | | | v | | | | | | | | - | | | | | | | | | | | | | | v | | | | | | | | | | | | |  | | | | | | | |  | | | | | |  | | | | | |  | |
| ST82 | - | - | - | v | v | | | | v | | | | | | | | v | | | | | | | | | | | | | | - | | | | | | | | | | | | |  | | | | | | | |  | | | | | |  | | | | | |  | |
| ST83 | - | - | v | v | v | | | | v | | | | | | | | v | | | | | | | | | | | | | | - | | | | | | | | | | | | | *nusA* PA4745 | | | | | | | | | | | | | | Transcripion elongation factor | | | | | |  | |
| ST84 | - | - | - | v | v | | | | v | | | | | | | | - | | | | | | | | | | | | | | - | | | | | | | | | | | | |  | | | | | | | |  | | | | | |  | | | | | |  | |
| ST85 | v | v | v | v | v | | | | - | | | | | | | | v | | | | | | | | | | | | | | - | | | | | | | | | | | | |  | | | | | | | |  | | | | | |  | | | | | |  | |
| ST86 | v | v | - | - | - | | | | - | | | | | | | | - | | | | | | | | | | | | | | - | | | | | | | | | | | | |  | | | | | | | |  | | | | | |  | | | | | |  | |
| ST87 | - | - | v | v | - | | | | - | | | | | | | | - | | | | | | | | | | | | | | v | | | | | | | | | | | | |  | | | | | | | |  | | | | | |  | | | | | |  | |
| ST88 | - | - | - | - | v | | | | v | | | | | | | | - | | | | | | | | | | | | | | - | | | | | | | | | | | | | *nirN* | | | | | | | | PA0509 | | | | | | probable c-type cytochrome NirN, PA0509 | | | | | |  | |
| ST89 | - | - | v | v | v | | | | v | | | | | | | | v | | | | | | | | | | | | | | v | | | | | | | | | | | | |  | | | | | | | |  | | | | | |  | | | | | |  | |
| ST9 | v | v | v | v | v | | | | v | | | | | | | | v | | | | | | | | | | | | | | - | | | | | | | | | | | | |  | | | | | | | |  | | | | | |  | | | | | |  | |
| ST90 | v | v | v | - | v | | | | v | | | | | | | | v | | | | | | | | | | | | | | v | | | | | | | | | | | | | *fusA1/fusA2* | | | | | | | | PA4266/2071 | | | | | | elongation factor G | | | | | |  | |
| ST91 | v | - | - | - | v | | | | v | | | | | | | | v | | | | | | | | | | | | | | - | | | | | | | | | | | | |  | | | | | | | |  | | | | | |  | | | | | |  | |
| ST92 | v | v | v | - | v | | | | v | | | | | | | | v | | | | | | | | | | | | | | v | | | | | | | | | | | | |  | | | | | | | |  | | | | | |  | | | | | |  | |
| ST93 | - | - | - | - | v | | | | v | | | | | | | | - | | | | | | | | | | | | | | - | | | | | | | | | | | | |  | | | | | | | |  | | | | | |  | | | | | |  | |
| ST94 | - | v | v | v | v | | | | v | | | | | | | | v | | | | | | | | | | | | | | v | | | | | | | | | | | | |  | | | | | | | |  | | | | | |  | | | | | |  | |
| ST95 | - | - | - | - | v | | | | v | | | | | | | | - | | | | | | | | | | | | | | - | | | | | | | | | | | | |  | | | | | | | |  | | | | | |  | | | | | |  | |
| ST96 | - | - | - | - | v | | | | v | | | | | | | | - | | | | | | | | | | | | | | v | | | | | | | | | | | | |  | | | | | | | |  | | | | | |  | | | | | |  | |
| ST97 | - | v | v | - | - | | | | - | | | | | | | | v | | | | | | | | | | | | | | v | | | | | | | | | | | | |  | | | | | | | |  | | | | | |  | | | | | |  | |
| ST98 | v | v | v | v | - | | | | - | | | | | | | | v | | | | | | | | | | | | | | v | | | | | | | | | | | | |  | | | | | | | |  | | | | | |  | | | | | |  | |
| ST99 | v | - | - | - | v | | | | v | | | | | | | | - | | | | | | | | | | | | | | v | | | | | | | | | | | | |  | | | | | | | |  | | | | | |  | | | | | |  | |
| ST101 | - | - | - | v | v | | | | - | | | | | | | | - | | | | | | | | | | | | | | - | | | | | | | | | | | | |  | | | | | | | |  | | | | | |  | | | | | |  | |
| ST102 | - | - | - | - | v | | | | v | | | | | | | | - | | | | | | | | | | | | | | v | | | | | | | | | | | | |  | | | | | | | |  | | | | | |  | | | | | |  | |
| ST103 | - | - | - | v | v | | | | - | | | | | | | | - | | | | | | | | | | | | | | v | | | | | | | | | | | | |  | | | | | | | |  | | | | | |  | | | | | |  | |
| ST104 | - | - | - | v | v | | | | v | | | | | | | | - | | | | | | | | | | | | | | v | | | | | | | | | | | | |  | | | | | | | |  | | | | | |  | | | | | |  | |
| ST105 | - | - | - | - | v | | | | v | | | | | | | | - | | | | | | | | | | | | | | v | | | | | | | | | | | | |  | | | | | | | |  | | | | | |  | | | | | |  | |
| ST106 | - | - | - | v | v | | | | v | | | | | | | | v | | | | | | | | | | | | | | v | | | | | | | | | | | | |  | | | | | | | | PA5312 | | | | | | probable aldehyde dehydrogenase | | | | | |  | |
| ST107 | - | - | - | v | v | | | | v | | | | | | | | v | | | | | | | | | | | | | | v | | | | | | | | | | | | | *gdhB* | | | | | | | | PA3068 | | | | | | NAD-dependent glutamate dehydrogenase | | | | | |  | |
| ST108 | - | - | - | v | v | | | | v | | | | | | | | v | | | | | | | | | | | | | | v | | | | | | | | | | | | |  | | | | | | | |  | | | | | |  | | | | | |  | |
| ST109 | - | - | - | - | v | | | | v | | | | | | | | **-** | | | | | | | | | | | | | | **v** | | | | | | | | | | | | | *accC* | | | | | | | | PA4848 | | | | | | biotin carboxylase | | | | | |  | |
| ST110 | v | v | v | - | - | | | | - | | | | | | | | **v** | | | | | | | | | | | | | | **-** | | | | | | | | | | | | |  | | | | | | | |  | | | | | |  | | | | | |  | |
| **8 hrs-specific phosphorylated 2D-proteins** | | | | | |  | |  | | |  | |  | | |  | | | | |  | | |  | | | | | | | | |  | | | | | | | | | | | | |  | | | | | | |  | | | | | |  | | | | |  | |
| ST111 | - | v | - | - | - | | | | - | | | | | | | | **-** | | | | | | | | | | | | | | **-** | | | | | | | | | | | | |  | | | | | | | |  | | | | | |  | | | | | |  | |
| ST112 | - | v | - | - | - | | | | - | | | | | | | | **-** | | | | | | | | | | | | | | **-** | | | | | | | | | | | | |  | | | | | | | |  | | | | | | | | | | | |  | |
| ST113 | - | v | - | - | - | | | | - | | | | | | | | **v** | | | | | | | | | | | | | | **-** | | | | | | | | | | | | |  | | | | | | | |  | | | | | |  | | | | | |  | |
| ST114 | - | v | - | - | - | | | | - | | | | | | | | **v** | | | | | | | | | | | | | | **-** | | | | | | | | | | | | |  | | | | | | | |  | | | | | |  | | | | | |  | |
| ST115 | - | v | - | - | - | | | | - | | | | | | | | **v** | | | | | | | | | | | | | | **-** | | | | | | | | | | | | |  | | | | | | | |  | | | | | |  | | | | | |  | |
| ST116 | - | v | - | - | - | | | | - | | | | | | | | **v** | | | | | | | | | | | | | | **-** | | | | | | | | | | | | |  | | | | | | | |  | | | | | |  | | | | | |  | |
| ST117 | - | v | - | - | - | | | | - | | | | | | | | **-** | | | | | | | | | | | | | | **-** | | | | | | | | | | | | |  | | | | | | | |  | | | | | |  | | | | | |  | |
| ST118 | - | v | - | - | - | | | | - | | | | | | | | **v** | | | | | | | | | | | | | | **-** | | | | | | | | | | | | |  | | | | | | | |  | | | | | |  | | | | | |  | |
| ST119 | - | v | - | - | - | | | | - | | | | | | | | **-** | | | | | | | | | | | | | | **-** | | | | | | | | | | | | |  | | | | | | | |  | | | | | |  | | | | | |  | |
| ST120 | - | v | - | - | - | | | | - | | | | | | | | **v** | | | | | | | | | | | | | | **-** | | | | | | | | | | | | |  | | | | | | | |  | | | | | |  | | | | | |  | |
| ST121 | - | v | - | - | - | | | | - | | | | | | | | **v** | | | | | | | | | | | | | | **-** | | | | | | | | | | | | |  | | | | | | | |  | | | | | |  | | | | | |  | |
| ST122 | - | v | - | - | - | | | | - | | | | | | | | **v** | | | | | | | | | | | | | | **-** | | | | | | | | | | | | |  | | | | | | | |  | | | | | |  | | | | | |  | |
| ST123 | - | v | - | - | - | | | | - | | | | | | | | **-** | | | | | | | | | | | | | | **-** | | | | | | | | | | | | |  | | | | | | | |  | | | | | |  | | | | | |  | |
| ST124 | - | v | - | - | - | | | | - | | | | | | | | **v** | | | | | | | | | | | | | | **-** | | | | | | | | | | | | |  | | | | | | | |  | | | | | |  | | | | | |  | |
| ST125 | - | v | - | - | - | | | | - | | | | | | | | **-** | | | | | | | | | | | | | | **-** | | | | | | | | | | | | |  | | | | | | | |  | | | | | |  | | | | | |  | |
| ST126 | - | v | - | - | - | | | | - | | | | | | | | **v** | | | | | | | | | | | | | | **-** | | | | | | | | | | | | |  | | | | | | | |  | | | | | |  | | | | | |  | |
| ST127 | - | v | - | - | - | | | | - | | | | | | | | **v** | | | | | | | | | | | | | | **-** | | | | | | | | | | | | |  | | | | | | | |  | | | | | |  | | | | | |  | |
| ST130 | - | v | - | - | - | | | | - | | | | | | | | **-** | | | | | | | | | | | | | | **-** | | | | | | | | | | | | |  | | | | | | | |  | | | | | |  | | | | | |  | |
| ST131 | - | v | - | - | - | | | | - | | | | | | | | **v** | | | | | | | | | | | | | | **-** | | | | | | | | | | | | |  | | | | | | | |  | | | | | |  | | | | | |  | |
| ST132 | - | v | - | - | - | | | | - | | | | | | | | **v** | | | | | | | | | | | | | | **-** | | | | | | | | | | | | |  | | | | | | | |  | | | | | |  | | | | | |  | |
| ST133 | - | v | - | - | - | | | | - | | | | | | | | **v** | | | | | | | | | | | | | | **-** | | | | | | | | | | | | |  | | | | | | | |  | | | | | |  | | | | | |  | |
| ST134 | - | v | - | - | - | | | | - | | | | | | | | **-** | | | | | | | | | | | | | | **-** | | | | | | | | | | | | |  | | | | | | | |  | | | | | |  | | | | | |  | |
| ST135 | - | v | - | - | - | | | | - | | | | | | | | **-** | | | | | | | | | | | | | | **-** | | | | | | | | | | | | |  | | | | | | | |  | | | | | |  | | | | | |  | |
| **24-hour-specific phosphorylated 2D-proteins** | | | | | | | | | | | | |  | | | | |  | | |  | | | |  | | | |  | | | | |  | | | |  | | | |  | | | |  | | | | | | | |  | | | | | |  | | | | |  |
| ST136 | - | - | v | - | - | | | | - | | | | | | | | **-** | | | | | | | | | | | | | | **-** | | | | | | | | | | | | |  | | | | | | | |  | | | | | |  | | | | | |  | |
| ST137 | - | - | v | - | - | | | | - | | | | | | | | **-** | | | | | | | | | | | | | | **-** | | | | | | | | | | | | |  | | | | | | | | | | | | |  | | | | | | |  | |
| ST138 | - | - | v | - | - | | | | - | | | | | | | | **-** | | | | | | | | | | | | | | **-** | | | | | | | | | | | | |  | | | | | | | |  | | | | | |  | | | | | |  | |
| ST139 | - | - | v | - | - | | | | - | | | | | | | | **-** | | | | | | | | | | | | | | **-** | | | | | | | | | | | | |  | | | | | | | |  | | | | | |  | | | | | |  | |
| ST140 | - | - | v | - | - | | | | - | | | | | | | | **-** | | | | | | | | | | | | | | **-** | | | | | | | | | | | | |  | | | | | | | |  | | | | | |  | | | | | |  | |
| ST142 | - | - | v | - | - | | | | - | | | | | | | | **-** | | | | | | | | | | | | | | **-** | | | | | | | | | | | | |  | | | | | | | |  | | | | | |  | | | | | |  | |
| ST143 | - | - | v | - | - | | | | - | | | | | | | | **-** | | | | | | | | | | | | | | **-** | | | | | | | | | | | | |  | | | | | | | |  | | | | | |  | | | | | |  | |
| ST144 | - | - | v | - | - | | | | - | | | | | | | | **-** | | | | | | | | | | | | | | **-** | | | | | | | | | | | | |  | | | | | | | |  | | | | | |  | | | | | |  | |
| ST145 | - | - | v | - | - | | | | - | | | | | | | | **-** | | | | | | | | | | | | | | **-** | | | | | | | | | | | | |  | | | | | | | |  | | | | | |  | | | | | |  | |
| ST146 | - | - | v | - | - | | | | - | | | | | | | | **-** | | | | | | | | | | | | | | **-** | | | | | | | | | | | | |  | | | | | | | |  | | | | | |  | | | | | |  | |
| ST148 | - | - | v | - | - | | | | - | | | | | | | | **-** | | | | | | | | | | | | | | **-** | | | | | | | | | | | | |  | | | | | | | |  | | | | | |  | | | | | |  | |
| ST150 | - | - | v | - | - | | | | - | | | | | | | | **-** | | | | | | | | | | | | | | **-** | | | | | | | | | | | | |  | | | | | | | |  | | | | | |  | | | | | |  | |
| ST151 | - | - | v | - | - | | | | - | | | | | | | | **-** | | | | | | | | | | | | | | **-** | | | | | | | | | | | | |  | | | | | | | |  | | | | | |  | | | | | |  | |
| ST152 | - | - | v | - | - | | | | - | | | | | | | | **-** | | | | | | | | | | | | | | **-** | | | | | | | | | | | | |  | | | | | | | |  | | | | | |  | | | | | |  | |
| ST154 | - | - | v | - | - | | | | - | | | | | | | | **-** | | | | | | | | | | | | | | **-** | | | | | | | | | | | | |  | | | | | | | |  | | | | | |  | | | | | |  | |
| ST155 | - | - | v | - | - | | | | - | | | | | | | | **-** | | | | | | | | | | | | | | **-** | | | | | | | | | | | | |  | | | | | | | |  | | | | | |  | | | | | |  | |
| ST156 | - | - | v | - | - | | | | - | | | | | | | | **-** | | | | | | | | | | | | | | **-** | | | | | | | | | | | | |  | | | | | | | |  | | | | | |  | | | | | |  | |
| ST158 | - | - | v | - | - | | | | - | | | | | | | | **v** | | | | | | | | | | | | | | **-** | | | | | | | | | | | | |  | | | | | | | |  | | | | | |  | | | | | |  | |
| ST159 | - | - | v | - | - | | | | - | | | | | | | | **-** | | | | | | | | | | | | | | **-** | | | | | | | | | | | | |  | | | | | | | |  | | | | | |  | | | | | |  | |
| ST160 | - | - | v | - | - | | | | - | | | | | | | | **v** | | | | | | | | | | | | | | **-** | | | | | | | | | | | | |  | | | | | | | |  | | | | | |  | | | | | |  | |
| ST161 | - | - | v | - | - | | | | - | | | | | | | | **-** | | | | | | | | | | | | | | **-** | | | | | | | | | | | | |  | | | | | | | |  | | | | | |  | | | | | |  | |
| **72-hour-specific phosphorylated 2D-proteins** | | | | | | | | | | | | | | | | | | | | | | | | | | | |  | | | |  | | | |  | | | |  | | |  | | | | |  |  |  |  | | | | |  | | | | | |  | | | |
| ST162 | - | - | - | v | - | | | | - | | | | | | | | **-** | | | | | | | | | | | | | | **v** | | | | | | | | | | | | |  | | | | | | | |  | | | | | |  | | | | | |  | |
| ST163 | - | - | - | v | - | | | | - | | | | | | | | **-** | | | | | | | | | | | | | | **-** | | | | | | | | | | | | |  | | | | | | | | | | | | |  | | | | | | |  | |
| ST164 | - | - | - | v | - | | | | - | | | | | | | | **-** | | | | | | | | | | | | | | **-** | | | | | | | | | | | | |  | | | | | | | |  | | | | | |  | | | | | |  | |
| ST165 | - | - | - | v | - | | | | - | | | | | | | | **-** | | | | | | | | | | | | | | **-** | | | | | | | | | | | | |  | | | | | | | |  | | | | | |  | | | | | |  | |
| ST167 | - | - | - | v | - | | | | - | | | | | | | | **-** | | | | | | | | | | | | | | **v** | | | | | | | | | | | | |  | | | | | | | |  | | | | | |  | | | | | |  | |
| ST168 | - | - | - | v | - | | | | - | | | | | | | | **-** | | | | | | | | | | | | | | **-** | | | | | | | | | | | | |  | | | | | | | |  | | | | | |  | | | | | |  | |
| ST169 | - | - | - | v | - | | | | - | | | | | | | | **-** | | | | | | | | | | | | | | **v** | | | | | | | | | | | | |  | | | | | | | |  | | | | | |  | | | | | |  | |
| ST170 | - | - | - | v | - | | | | - | | | | | | | | **-** | | | | | | | | | | | | | | **-** | | | | | | | | | | | | |  | | | | | | | |  | | | | | |  | | | | | |  | |
| ST172 | - | - | - | v | - | | | | - | | | | | | | | **-** | | | | | | | | | | | | | | **-** | | | | | | | | | | | | |  | | | | | | | |  | | | | | |  | | | | | |  | |
| ST173 | - | - | - | v | - | | | | - | | | | | | | | **-** | | | | | | | | | | | | | | **-** | | | | | | | | | | | | |  | | | | | | | |  | | | | | |  | | | | | |  | |
| ST174 | - | - | - | v | - | | | | - | | | | | | | | **-** | | | | | | | | | | | | | | **-** | | | | | | | | | | | | |  | | | | | | | |  | | | | | |  | | | | | |  | |
| ST175 | - | - | - | v | - | | | | - | | | | | | | | **-** | | | | | | | | | | | | | | **v** | | | | | | | | | | | | |  | | | | | | | |  | | | | | |  | | | | | |  | |
| ST176 | - | - | - | v | - | | | | - | | | | | | | | **-** | | | | | | | | | | | | | | **-** | | | | | | | | | | | | |  | | | | | | | |  | | | | | |  | | | | | |  | |
| ST177 | - | - | - | v | - | | | | - | | | | | | | | **-** | | | | | | | | | | | | | | **-** | | | | | | | | | | | | |  | | | | | | | |  | | | | | |  | | | | | |  | |
| ST178 | - | - | - | v | - | | | | - | | | | | | | | **-** | | | | | | | | | | | | | | **v** | | | | | | | | | | | | |  | | | | | | | |  | | | | | |  | | | | | |  | |
| ST179 | - | - | - | v | - | | | | - | | | | | | | | **-** | | | | | | | | | | | | | | **-** | | | | | | | | | | | | |  | | | | | | | |  | | | | | |  | | | | | |  | |
| ST180 | - | - | - | v | - | | | | - | | | | | | | | **-** | | | | | | | | | | | | | | **-** | | | | | | | | | | | | |  | | | | | | | |  | | | | | |  | | | | | |  | |
| ST181 | - | - | - | v | - | | | | - | | | | | | | | **-** | | | | | | | | | | | | | | **-** | | | | | | | | | | | | |  | | | | | | | |  | | | | | |  | | | | | |  | |
| ST182 | - | - | - | v | - | | | | - | | | | | | | | **-** | | | | | | | | | | | | | | **-** | | | | | | | | | | | | |  | | | | | | | |  | | | | | |  | | | | | |  | |
| ST183 | - | - | - | v | - | | | | - | | | | | | | | **-** | | | | | | | | | | | | | | **-** | | | | | | | | | | | | |  | | | | | | | |  | | | | | |  | | | | | |  | |
| ST184 | - | - | - | v | - | | | | - | | | | | | | | **-** | | | | | | | | | | | | | | **-** | | | | | | | | | | | | |  | | | | | | | |  | | | | | |  | | | | | |  | |
| ST185 | - | - | - | v | - | | | | - | | | | | | | | **-** | | | | | | | | | | | | | | **-** | | | | | | | | | | | | |  | | | | | | | |  | | | | | |  | | | | | |  | |
| ST186 | - | - | - | v | - | | | | - | | | | | | | | **-** | | | | | | | | | | | | | | **-** | | | | | | | | | | | | |  | | | | | | | |  | | | | | |  | | | | | |  | |
| ST187 | - | - | - | v | - | | | | - | | | | | | | | **-** | | | | | | | | | | | | | | **v** | | | | | | | | | | | | |  | | | | | | | |  | | | | | |  | | | | | |  | |
| ST188 | - | - | - | v | - | | | | - | | | | | | | | **-** | | | | | | | | | | | | | | **v** | | | | | | | | | | | | |  | | | | | | | |  | | | | | |  | | | | | |  | |
| ST189 | - | - | - | v | - | | | | - | | | | | | | | **-** | | | | | | | | | | | | | | **-** | | | | | | | | | | | | |  | | | | | | | |  | | | | | |  | | | | | |  | |
| ST190 | - | - | - | v | - | | | | - | | | | | | | | **-** | | | | | | | | | | | | | | **v** | | | | | | | | | | | | |  | | | | | | | |  | | | | | |  | | | | | |  | |
| **144-hour-specific phosphorylated 2D-proteins** | | | | | | |  | | |  | | | |  | | | | |  | | |  | | | |  | | | |  | | | |  | | | | | | | | | | | | |  | | | | | | | |  | | | | | |  | | |  | |
| ST191 | - | - | - | - | v | | | | - | | | | | | | | **-** | | | | | | | | | | | | | | **-** | | | | | | | | | | | | |  | | | | | | | |  | | | | | |  | | | | | |  | |
| ST192 | - | - | - | - | v | | | | - | | | | | | | | **-** | | | | | | | | | | | | | | **v** | | | | | | | | | | | | |  | | | | | | | | | | | | |  | | | | | | |  | |
| ST193 | - | - | - | - | v | | | | - | | | | | | | | **-** | | | | | | | | | | | | | | **v** | | | | | | | | | | | | |  | | | | | | | |  | | | | | |  | | | | | |  | |
| ST194 | - | - | - | - | v | | | | - | | | | | | | | **-** | | | | | | | | | | | | | | **-** | | | | | | | | | | | | |  | | | | | | | |  | | | | | |  | | | | | |  | |
| ST195 | - | - | - | - | v | | | | - | | | | | | | | **-** | | | | | | | | | | | | | | **v** | | | | | | | | | | | | |  | | | | | | | |  | | | | | |  | | | | | |  | |
| ST196 | - | - | - | - | v | | | | - | | | | | | | | **-** | | | | | | | | | | | | | | **v** | | | | | | | | | | | | |  | | | | | | | |  | | | | | |  | | | | | |  | |
| ST197 | - | - | - | - | v | | | | - | | | | | | | | **-** | | | | | | | | | | | | | | **-** | | | | | | | | | | | | |  | | | | | | | |  | | | | | |  | | | | | |  | |
| ST198 | - | - | - | - | v | | | | - | | | | | | | | **-** | | | | | | | | | | | | | | **-** | | | | | | | | | | | | |  | | | | | | | |  | | | | | |  | | | | | |  | |
| ST199 | - | - | - | - | v | | | | - | | | | | | | | **-** | | | | | | | | | | | | | | **v** | | | | | | | | | | | | |  | | | | | | | |  | | | | | |  | | | | | |  | |
| ST200 | - | - | - | - | v | | | | - | | | | | | | | **-** | | | | | | | | | | | | | | **-** | | | | | | | | | | | | |  | | | | | | | |  | | | | | |  | | | | | |  | |
| ST201 | - | - | - | - | v | | | | - | | | | | | | | **-** | | | | | | | | | | | | | | **-** | | | | | | | | | | | | |  | | | | | | | |  | | | | | |  | | | | | |  | |
| ST202 | - | - | - | - | v | | | | - | | | | | | | | **-** | | | | | | | | | | | | | | **v** | | | | | | | | | | | | |  | | | | | | | |  | | | | | |  | | | | | |  | |
| ST203 | - | - | - | - | v | | | | - | | | | | | | | **-** | | | | | | | | | | | | | | **-** | | | | | | | | | | | | |  | | | | | | | |  | | | | | |  | | | | | |  | |
| ST204 | - | - | - | - | v | | | | - | | | | | | | | **-** | | | | | | | | | | | | | | **-** | | | | | | | | | | | | |  | | | | | | | |  | | | | | |  | | | | | |  | |
| ST205 | - | - | - | - | v | | | | - | | | | | | | | **-** | | | | | | | | | | | | | | **v** | | | | | | | | | | | | |  | | | | | | | |  | | | | | |  | | | | | |  | |
| ST206 | - | - | - | - | v | | | | - | | | | | | | | **-** | | | | | | | | | | | | | | **-** | | | | | | | | | | | | |  | | | | | | | |  | | | | | |  | | | | | |  | |
| ST207 | - | - | - | - | v | | | | - | | | | | | | | **-** | | | | | | | | | | | | | | **v** | | | | | | | | | | | | |  | | | | | | | |  | | | | | |  | | | | | |  | |
| ST208 | - | - | - | - | v | | | | - | | | | | | | | **-** | | | | | | | | | | | | | | **v** | | | | | | | | | | | | |  | | | | | | | |  | | | | | |  | | | | | |  | |
| ST209 | - | - | - | - | v | | | | - | | | | | | | | **-** | | | | | | | | | | | | | | **-** | | | | | | | | | | | | |  | | | | | | | |  | | | | | |  | | | | | |  | |
| ST210 | - | - | - | - | v | | | | - | | | | | | | | **-** | | | | | | | | | | | | | | **v** | | | | | | | | | | | | |  | | | | | | | |  | | | | | |  | | | | | |  | |
| ST211 | - | - | - | - | v | | | | - | | | | | | | | **-** | | | | | | | | | | | | | | **-** | | | | | | | | | | | | |  | | | | | | | |  | | | | | |  | | | | | |  | |
| ST212 | - | - | - | - | v | | | | - | | | | | | | | **-** | | | | | | | | | | | | | | **v** | | | | | | | | | | | | |  | | | | | | | |  | | | | | |  | | | | | |  | |
| ST213 | - | - | - | - | v | | | | - | | | | | | | | **-** | | | | | | | | | | | | | | **-** | | | | | | | | | | | | |  | | | | | | | |  | | | | | |  | | | | | |  | |
| ST214 | - | - | - | - | v | | | | - | | | | | | | | **-** | | | | | | | | | | | | | | **-** | | | | | | | | | | | | |  | | | | | | | |  | | | | | |  | | | | | |  | |
| ST216 | - | - | - | - | v | | | | - | | | | | | | | **-** | | | | | | | | | | | | | | **v** | | | | | | | | | | | | |  | | | | | | | |  | | | | | |  | | | | | |  | |
| ST217 | - | - | - | - | v | | | | - | | | | | | | | **-** | | | | | | | | | | | | | | **v** | | | | | | | | | | | | |  | | | | | | | |  | | | | | |  | | | | | |  | |
| ST218 | - | - | - | - | v | | | | - | | | | | | | | **-** | | | | | | | | | | | | | | **-** | | | | | | | | | | | | |  | | | | | | | |  | | | | | |  | | | | | |  | |
| ST219 | - | - | - | - | v | | | | - | | | | | | | | **-** | | | | | | | | | | | | | | **-** | | | | | | | | | | | | |  | | | | | | | |  | | | | | |  | | | | | |  | |
| ST220 | - | - | - | - | v | | | | - | | | | | | | | **-** | | | | | | | | | | | | | | **v** | | | | | | | | | | | | |  | | | | | | | |  | | | | | |  | | | | | |  | |
| ST221 | - | - | - | - | v | | | | - | | | | | | | | **-** | | | | | | | | | | | | | | **-** | | | | | | | | | | | | |  | | | | | | | |  | | | | | |  | | | | | |  | |
| ST222 | - | - | - | - | v | | | | - | | | | | | | | **-** | | | | | | | | | | | | | | **-** | | | | | | | | | | | | |  | | | | | | | |  | | | | | |  | | | | | |  | |
| ST223 | - | - | - | - | v | | | | - | | | | | | | | **-** | | | | | | | | | | | | | | **v** | | | | | | | | | | | | |  | | | | | | | |  | | | | | |  | | | | | |  | |
| ST224 | - | - | - | - | v | | | | - | | | | | | | | **-** | | | | | | | | | | | | | | **-** | | | | | | | | | | | | |  | | | | | | | |  | | | | | |  | | | | | |  | |
| ST225 | - | - | - | - | v | | | | - | | | | | | | | **-** | | | | | | | | | | | | | | **v** | | | | | | | | | | | | |  | | | | | | | |  | | | | | |  | | | | | |  | |
| ST226 | - | - | - | - | v | | | | - | | | | | | | | **-** | | | | | | | | | | | | | | **-** | | | | | | | | | | | | |  | | | | | | | |  | | | | | |  | | | | | |  | |
| ST227 | - | - | - | - | v | | | | - | | | | | | | | **-** | | | | | | | | | | | | | | **-** | | | | | | | | | | | | |  | | | | | | | |  | | | | | |  | | | | | |  | |
| **216-hour-specific phosphorylated 2D-proteins** | | | | | | |  | | |  | | | |  | | | | |  | | |  | | | |  | | | |  | | | |  | | | | | | | | | | | | |  | | | | | | | |  | | | | | |  | | |  | |
| ST228 | - | - | - | - | - | | | | v | | | | | | | | **-** | | | | | | | | | | | | | | **-** | | | | | | | | | | | | |  | | | | | | | |  | | | | | |  | | | | | |  | |
| ST229 | - | - | - | - | - | | | | v | | | | | | | | **-** | | | | | | | | | | | | | | **-** | | | | | | | | | | | | |  | | | | | | | |  | | | | | |  | | | | | |  | |
| ST230 | - | - | - | - | - | | | | v | | | | | | | | **-** | | | | | | | | | | | | | | **-** | | | | | | | | | | | | |  | | | | | | | |  | | | | | |  | | | | | |  | |
| ST231 | - | - | - | - | - | | | | v | | | | | | | | **-** | | | | | | | | | | | | | | **-** | | | | | | | | | | | | |  | | | | | | | |  | | | | | |  | | | | | |  | |
| ST232 | - | - | - | - | - | | | | v | | | | | | | | **-** | | | | | | | | | | | | | | **-** | | | | | | | | | | | | |  | | | | | | | |  | | | | | |  | | | | | |  | |
| ST233 | - | - | - | - | - | | | | v | | | | | | | | **-** | | | | | | | | | | | | | | **-** | | | | | | | | | | | | |  | | | | | | | |  | | | | | |  | | | | | |  | |
| ST234 | - | - | - | - | - | | | | v | | | | | | | | **-** | | | | | | | | | | | | | | **-** | | | | | | | | | | | | |  | | | | | | | |  | | | | | |  | | | | | |  | |
| ST235 | - | - | - | - | - | | | | v | | | | | | | | **-** | | | | | | | | | | | | | | **-** | | | | | | | | | | | | |  | | | | | | | |  | | | | | |  | | | | | |  | |
| ST236 | - | - | - | - | - | | | | v | | | | | | | | **-** | | | | | | | | | | | | | | **-** | | | | | | | | | | | | |  | | | | | | | |  | | | | | |  | | | | | |  | |
| ST237 | - | - | - | - | - | | | | v | | | | | | | | **-** | | | | | | | | | | | | | | **-** | | | | | | | | | | | | |  | | | | | | | | PA0799 | | | | | | probable helicase | | | | | |  | |
| ST238 | - | - | - | - | - | | | | v | | | | | | | | **-** | | | | | | | | | | | | | | **-** | | | | | | | | | | | | |  | | | | | | | |  | | | | | |  | | | | | |  | |
| ST239 | - | - | - | - | - | | | | v | | | | | | | | **-** | | | | | | | | | | | | | | **-** | | | | | | | | | | | | |  | | | | | | | |  | | | | | |  | | | | | |  | |
| ST240 | - | - | - | - | - | | | | v | | | | | | | | **-** | | | | | | | | | | | | | | **-** | | | | | | | | | | | | |  | | | | | | | |  | | | | | |  | | | | | |  | |
| ST241 | - | - | - | - | - | | | | v | | | | | | | | **-** | | | | | | | | | | | | | | **-** | | | | | | | | | | | | |  | | | | | | | |  | | | | | |  | | | | | |  | |
| ST242 | - | - | - | - | - | | | | v | | | | | | | | **-** | | | | | | | | | | | | | | **-** | | | | | | | | | | | | |  | | | | | | | |  | | | | | |  | | | | | |  | |
| ST243 | - | - | - | - | - | | | | v | | | | | | | | **-** | | | | | | | | | | | | | | **-** | | | | | | | | | | | | |  | | | | | | | |  | | | | | |  | | | | | |  | |
| ST244 | - | - | - | - | - | | | | v | | | | | | | | **-** | | | | | | | | | | | | | | **-** | | | | | | | | | | | | |  | | | | | | | |  | | | | | |  | | | | | |  | |
| ST245 | - | - | - | - | - | | | | v | | | | | | | | **-** | | | | | | | | | | | | | | **-** | | | | | | | | | | | | |  | | | | | | | |  | | | | | |  | | | | | |  | |
| ST246 | - | - | - | - | - | | | | v | | | | | | | | **-** | | | | | | | | | | | | | | **-** | | | | | | | | | | | | |  | | | | | | | |  | | | | | |  | | | | | |  | |
| ST247 | - | - | - | - | - | | | | v | | | | | | | | **-** | | | | | | | | | | | | | | **-** | | | | | | | | | | | | |  | | | | | | | |  | | | | | |  | | | | | |  | |
| **Planktonic-specific phosphorylated 2D-proteins** | | | | | | | | | | | | |  | | |  | | | | |  | | |  | | | |  | | | | |  | | | |  | | | |  | | | |  | | | | | | | | | | | |  | | | | | | |  | |
| ST248 | v | - | - | - | - | | | | - | | | | | | | | **-** | | | | | | | | | | | | | | **-** | | | | | | | | | | | | |  | | | | | | | | | | | | |  | | | | | | |  | |
| ST249 | v | - | - | - | - | | | | - | | | | | | | | **-** | | | | | | | | | | | | | | **-** | | | | | | | | | | | | |  | | | | | | | |  | | | | | |  | | | | | |  | |
| ST250 | v | - | - | - | - | | | | - | | | | | | | | **-** | | | | | | | | | | | | | | **-** | | | | | | | | | | | | |  | | | | | | | |  | | | | | |  | | | | | |  | |
| ST251 | v | - | - | - | - | | | | - | | | | | | | | **-** | | | | | | | | | | | | | | **-** | | | | | | | | | | | | |  | | | | | | | |  | | | | | |  | | | | | |  | |
| ST252 | v | - | - | - | - | | | | - | | | | | | | | **-** | | | | | | | | | | | | | | **-** | | | | | | | | | | | | |  | | | | | | | |  | | | | | |  | | | | | |  | |
| ST253 | v | - | - | - | - | | | | - | | | | | | | | **-** | | | | | | | | | | | | | | **-** | | | | | | | | | | | | |  | | | | | | | |  | | | | | |  | | | | | |  | |
| ST254 | v | - | - | - | - | | | | - | | | | | | | | **-** | | | | | | | | | | | | | | **-** | | | | | | | | | | | | |  | | | | | | | |  | | | | | |  | | | | | |  | |
| ST255 | v | - | - | - | - | | | | - | | | | | | | | **-** | | | | | | | | | | | | | | **-** | | | | | | | | | | | | |  | | | | | | | |  | | | | | |  | | | | | |  | |
| ST256 | v | - | - | - | - | | | | - | | | | | | | | **-** | | | | | | | | | | | | | | **-** | | | | | | | | | | | | |  | | | | | | | |  | | | | | |  | | | | | |  | |
| ST257 | v | - | - | - | - | | | | - | | | | | | | | **-** | | | | | | | | | | | | | | **-** | | | | | | | | | | | | |  | | | | | | | |  | | | | | |  | | | | | |  | |
| ST258 | v | - | - | - | - | | | | - | | | | | | | | **-** | | | | | | | | | | | | | | **-** | | | | | | | | | | | | |  | | | | | | | |  | | | | | |  | | | | | |  | |
| ST259 | v | - | - | - | - | | | | - | | | | | | | | **-** | | | | | | | | | | | | | | **-** | | | | | | | | | | | | |  | | | | | | | |  | | | | | |  | | | | | |  | |
| ST260 | v | - | - | - | - | | | | - | | | | | | | | **-** | | | | | | | | | | | | | | **-** | | | | | | | | | | | | |  | | | | | | | |  | | | | | |  | | | | | |  | |
| ST261 | v | - | - | - | - | | | | - | | | | | | | | **v** | | | | | | | | | | | | | | **-** | | | | | | | | | | | | |  | | | | | | | |  | | | | | |  | | | | | |  | |
| ST263 | v | - | - | - | - | | | | - | | | | | | | | **-** | | | | | | | | | | | | | | **-** | | | | | | | | | | | | |  | | | | | | | |  | | | | | |  | | | | | |  | |
| ST264 | v | - | - | - | - | | | | - | | | | | | | | **-** | | | | | | | | | | | | | | **-** | | | | | | | | | | | | |  | | | | | | | |  | | | | | |  | | | | | |  | |
| ST265 | v | - | - | - | - | | | | - | | | | | | | | **-** | | | | | | | | | | | | | | **-** | | | | | | | | | | | | |  | | | | | | | |  | | | | | |  | | | | | |  | |
| ST266 | v | - | - | - | - | | | | - | | | | | | | | **-** | | | | | | | | | | | | | | **-** | | | | | | | | | | | | |  | | | | | | | |  | | | | | |  | | | | | |  | |
| ST267 | v | - | - | - | - | | | | - | | | | | | | | **-** | | | | | | | | | | | | | | **-** | | | | | | | | | | | | |  | | | | | | | |  | | | | | |  | | | | | |  | |
| ST268 | v | - | - | - | - | | | | - | | | | | | | | **-** | | | | | | | | | | | | | | **-** | | | | | | | | | | | | |  | | | | | | | |  | | | | | |  | | | | | |  | |
| ST269 | V | - | - | - | - | | | | - | | | | | | | | **-** | | | | | | | | | | | | | | **-** | | | | | | | | | | | | |  | | | | | | | |  | | | | | |  | | | | | |  | |
| ST270 | V | - | - | - | - | | | | - | | | | | | | | **-** | | | | | | | | | | | | | | **-** | | | | | | | | | | | | |  | | | | | | | |  | | | | | |  | | | | | |  | |
| ST271 | V | - | - | - | - | | | | - | | | | | | | | **v** | | | | | | | | | | | | | | **-** | | | | | | | | | | | | |  | | | | | | | |  | | | | | |  | | | | | |  | |
| ST272 | V | - | - | - | - | | | | - | | | | | | | | **v** | | | | | | | | | | | | | | **-** | | | | | | | | | | | | |  | | | | | | | |  | | | | | |  | | | | | |  | |

*PL, planktonic cells; 8HR, 24HR, 72HR, 144HR, 216HR, biofilms grown for 8, 24, 72, 144, and 216 hours.

-, absent

V, present
