# Supplementary material for: A Novel Signaling Network Essential for Regulating Pseudomonas aeruginosa Biofilm Development
Source: PLoS Pathog. 2009 Nov 20;5(11):e1000668. doi: 10.1371/journal.ppat.1000668 (PMC2774163; doi:10.1371/journal.ppat.1000668)
Supplement: Table S3 — BLINK search for potential BifS, BfmR, and MifR homologues. (0.12 MB DOC) [file ppat.1000668.s007.doc]

Supplemental Table S3. BLINK search for potential BifS, BfmR, and MifR homologues.

| **Taxonomy** | **Species (strain)** | **BfmR** | | | **BfiS** | | | **MifR** | | |
| --- | --- | --- | --- | --- | --- | --- | --- | --- | --- | --- |
|  |  | **Accession (gi|)** | **Identity (%)** | **Expect** | **Accession (gi|)** | **Identity (%)** | **Expect** | **Accession (gi|)** | **Identity (%)** | **Expect** |
| Actinobacteria | *Nocardia farcinica* (IFM 10152) | - | - | - | 54024811 | 28 | 4E-29 | 54024806 | 43 | 2E-18 |
|  |  |  |  |  |  |  |  |  |  |  |
| Bacteroidetes | *Porphyromonas gingivalis* (ATCC 33277) | - | - | - | - | - | - | 188994639 | 45 | 5E-75 |
|  |  |  |  |  |  |  |  |  |  |  |
| Firmicutes | *Clostridium difficile* (630) | 126699276 | 35 | 5E-37 | - | - | - | 126699346 | 34 | 7E-74 |
|  | *Streptococcus pneumoniae* (TIGR4) | 15901898 | 38 | 2E-43 | - | - | - | - | - | - |
|  |  |  |  |  |  |  |  |  |  |  |
| Planctomycetes | *Gemmata obscuriglobus* (UQM 2246) | 168705023 | 39 | 1E-34 | 168700622 | 37 | 2E-41 | 168700124 | 40 | 3E-83 |
|  |  |  |  |  |  |  |  |  |  |  |
| Proteobacteria | *Methylobacterium extorquens* (PA1) | 163849959 | 79 | 4E-116 | 163852920 | 29 | 4E-41 | 163662293 | 54 | 1E-125 |
| Alpha | *Rhizobium leguminosarum* (bv. viciae 3841) | 116252485 | 81 | 7E-116 | 115254104 | 29 | 1E-37 | 115257816 | 52 | 4E-127 |
|  | *Sinorhizobium medicae* (WSM419) | 150376054 | 83 | 2E-121 | 150377612 | 29 | 2E-40 | 150376318 | 54 | 4E-128 |
|  | *Stappia aggregata* (IAM 12614) | 118593271 | 54 | 2E-63 | 118589373 | 28 | 2E-30 | 118591526 | 55 | 2E-136 |
|  | *Magnetospirillum magneticum* (AMB-1) | 82947284 | 54 | 7E-55 | 82944836 | 31 | 5E-38 | 82945596 | 60 | 3E-149 |
|  |  |  |  |  |  |  |  |  |  |  |
| Proteobacteria | *Burkholderia cenocepacia* (AU 1054) | 107025470 | 70 | 7E-99 | 107028682 | 35 | 1E-37 | 107023804 | 54 | 2E-124 |
| Beta | *Burkholderia dolosa* (AUO158) | 194565567 | 71 | 8E-99 | 194562940 | 35 | 4E-38 | 194562304 | 54 | 2E-125 |
|  | *Burkholderia multivorans* (CGD2M) | 221200233 | 70 | 5E-98 | 221199402 | 29 | 1E-45 | 221199909 | 54 | 9E-127 |
|  | *Chromobacterium violaceum* (ATCC 12472) | 34498562 | 49 | 1E-59 | 34497779 | 33 | 5E-46 | - | - | - |
|  |  |  |  |  |  |  |  |  |  |  |
| Proteobacteria | *Geobacter lovleyi (*SZ) | 189424148 | 50 | 1E-61 | - | - | - | 189424441 | 49 | 3E-87 |
| Delta | *Stigmatella aurantiaca* (DW4/3-1) | 115377047 | 75 | 4E-97 | - | - | - | 115380039 | 41 | 6E-90 |
|  | *Myxococcus xanthus (*DK 1622) | 108763038 | 43 | 7E-42 | - | - | - | 108758352 | 48 | 2E-88 |
|  |  |  |  |  |  |  |  |  |  |  |
| Proteobacteria | *Aeromonas hydrophila* (ATCC 7966) | 117621451 | 55 | 2E-68 | 117560473 | 31 | 1E-23 | 2160525 | 38 | 1E-36 |
| Gamma | *Enterobacter cancerogenus* (ATCC 35316) | 209908056 | 70 | 3E-94 | - | - | - | 209909741 | 50 | 1E-64 |
|  | *Escherichia coli (*O157:H7 str. Sakai) | 15834321 | 38 | 9E-39 | - | - | - | 15832674 | 40 | 5E-79 |
|  | *Klebsiella pneumoniae* (342) | 206578894 | 71 | 2E-101 | - | - | - | 206580994 | 58 | 6E-64 |
|  | *Yersinia intermedia* (ATCC 29909) | 77978323 | 45 | 2E-53 | 77976605 | 35 | 2E-31 | 77979534 | 50 | 6E-55 |
|  | *Azotobacter vinelandii* (DJ) | 226946662 | 45 | 1E-49 | 226944613 | 68 | 0 | 226943917 | 76 | 0 |
|  | *Pseudomonas putida* (F1) | 148548500 | 92 | 2E-127 | 148547443 | 60 | 1E-76 | 148549527 | 75 | 0 |
|  | *Pseudomonas entomophila* (L48) | 104781516 | 91 | 3E-126 | - | - | - | 104783313 | 74 | 0 |
|  | *Pseudomonas fluorescens* (Pf-5) | 70729143 | 55 | 8E-63 | 70729532 | 68 | 0 | 70732128 | 74 | 0 |
|  | *Pseudomonas mendocina* (ymp) | 146306182 | 49 | 3E-58 | 146308831 | 60 | 3E-127 | 146309575 | 73 | 0 |
|  | *Pseudomonas stutzeri (*A1501) | 146282783 | 48 | 6E-59 | - | - | - | 146280547 | 75 | 0 |
|  | *Pseudomonas syringae* (pv. tomato T1) | 213968160 | 56 | 2E-63 | 213968479 | 35 | 3E-32 | 213970254 | 72 | 0 |
|  | *Vibrio cholerae* (MZO-2) | 153825200 | 47 | 1E-52 | - | - | - | 153824995 | 48 | 7E-112 |
