# Supplementary material for: A Novel Signaling Network Essential for Regulating Pseudomonas aeruginosa Biofilm Development
Source: PLoS Pathog. 2009 Nov 20;5(11):e1000668. doi: 10.1371/journal.ppat.1000668 (PMC2774163; doi:10.1371/journal.ppat.1000668)
Supplement: Table S4 — Oligonucleotide sequences used for RT-PCR, cloning, and targeted gene inactivation. (0.04 MB DOC) [file ppat.1000668.s008.doc]

**Supplemental Table S4. Oligonucleotide sequences** used for RT-PCR, cloning, and targeted gene inactivation.

| **Oligonucleotide** | **Sequence** |
| --- | --- |
| ***RT-PCR*** | |
| PA4101RTf | GAAGCCAACACGGTG |
| PA4101RTr | CTGGTGTCCAGTTGC |
| PA4197RTf | CCTCAACAGCCTCCTG |
| PA4197RTr | CCATGCCATGCTCCAC |
| PA5511RTf | GTCGACGACGAAGCGAC |
| PA5511RTr | GCAACGCTTCCATCGGC |
| retSRTf | GGTACGGCTTCGGATC |
| retSRTr | GTAGGCGGGCTTCTC |
| ladSRTf | GGTGCGGTGAGCTTC |
| ladSRTr | CTGGATCGACCCCTG |
| pelGf | CCTCTACGCCGGGCTG |
| pelGr | CCAGCAGCAGGCCGTC |
| pslAf | GGCCTGGTTCCCGTGG |
| pslAr | CCGGTACAGGCGCAGC |
| mreBf | CTGTCGATCGACCTGGG |
| mreBr | CAGCCATCGGCTCTTCG |
| ***Inducible Expression*** | |
| PA4101pETf | CACCATGGAGCATGTCGATC |
| PA4101pET | TGGATGGGCCTCGACCAGTC |
| PA4101HisF | GCgaattcATGGAGCATGTCGATCAC |
| PA4101HisR | GGCactagtTCAATGGTGATGGTGATG |
| PA4197araF | GCGCGCgaattcATGAGTCCGCTTCCCGC |
| PA4197araR | GCGCGCtctagaTTACCCTCCCGTGGCGC |
| PA5511araF | GCGCgaattcATGAGCGACCAGGTG |
| PA5511araR | GCgagctcTTCAGGCCGGCTCTTC |
| ***Targeted Gene Inactivation**** | |
| PA5511f1 | CGgaattcGGCGGCGACTGGGTGC |
| PA5511r1 | CGggatccCGCGATCGACCGCCTG |
| PA5511f2 | CGggatccGCGAGCTGGCCAACGC |
| PA5511r2 | GCaagcttGTTCCCGCTGGTGGGC |

*, restriction sites are indicated by nucleotides in lower case
